# Developmental Ethanol Exposure Leads to Long-Term Deficits in Attention and Its Underlying Prefrontal Circuitry

**DOI:** 10.1523/ENEURO.0267-16.2016

**Published:** 2016-11-08

**Authors:** Emma L. Louth, Warren Bignell, Christine L. Taylor, Craig D.C. Bailey

**Affiliations:** Department of Biomedical Sciences, Ontario Veterinary College, University of Guelph, Guelph, Ontario N1G 2W1, Canada

**Keywords:** attention, developmental ethanol exposure, electrophysiology, fetal alcohol spectrum disorders, nicotinic receptors, prefrontal cortex

## Abstract

Chronic prenatal exposure to ethanol can lead to a spectrum of teratogenic outcomes that are classified in humans as fetal alcohol spectrum disorders (FASD). One of the most prevalent and persistent neurocognitive components of FASD is attention deficits, and it is now thought that these attention deficits differ from traditional attention deficit hyperactivity disorder (ADHD) in their quality and response to medication. However, the neuronal mechanisms underlying attention deficits in FASD are not well understood. We show here that after developmental binge-pattern ethanol exposure, adult mice exhibit impaired performance on the five-choice serial reaction time test for visual attention, with lower accuracy during initial training and a higher rate of omissions under challenging conditions of high attention demand. Whole-cell electrophysiology experiments in these same mice find dysregulated pyramidal neurons in layer VI of the medial prefrontal cortex, which are critical for normal attention performance. Layer VI neurons show decreased intrinsic excitability and increased responses to stimulation of both nicotinic acetylcholine receptors and α-amino-3-hydroxy-5-methyl-4-isoxazolepropionic acid (AMPA) glutamate receptors. Moreover, although nicotinic acetylcholine responses correlate with performance on the five-choice task in control mice, these relationships are completely disrupted in mice exposed to ethanol during development. These findings demonstrate a novel outcome of developmental binge-pattern ethanol exposure and suggest that persistent alterations to the function of prefrontal layer VI neurons play an important mechanistic role in attention deficits associated with FASD.

## Significance Statement

Children who exhibit fetal alcohol spectrum disorders (FASD) are often diagnosed with comorbid attention deficit hyperactivity disorder (ADHD), even though mechanisms underlying attention deficits in these two disorders are now believed to differ. We show in mice after developmental binge-pattern ethanol exposure that deficits on an attention task are accompanied by dysregulated function of prefrontal cortex layer VI pyramidal neurons, which are known to be critical for normal attention. These layer VI neurons show decreased intrinsic excitability and increased responses to excitatory neurotransmission, and relationships between their nicotinic signaling and attention performance are disrupted. These findings demonstrate novel mechanisms and potential therapeutic targets to mitigate attention deficits associated with FASD.

## Introduction

Chronic prenatal exposure to ethanol can lead to a spectrum of teratogenic outcomes in humans known collectively as fetal alcohol spectrum disorders (FASD; Sokol et al., 2003; Chudley et al., 2005; Riley et al., 2011). Potential manifestations of FASD include growth deficiency, specific craniofacial abnormalities, and persistent neurocognitive deficits (Chudley et al., 2005). The estimated prevalence of FASD ranges from approximately 31 to 34 per 1000 live births in the United States and Canada to 113 per 1000 live births in South Africa (Roozen et al., 2016), and this is known to impart significant costs to individuals and societies within their local education, judicial, and medical systems (Lupton et al., 2004; Popova et al., 2016). Deficits in attention rank among the most common and persistent neurocognitive components of FASD, for example, as a comorbid diagnosis of attention deficit hyperactivity disorder (ADHD) has been assigned to approximately 41–95% of children who are affected by FASD (Bhatara et al., 2006; Fryer et al., 2007). However, recent work suggests that the detailed pattern of attention deficits is distinct between these two disorders, including earlier onset and greater impairment to shifting attention in children affected by FASD (O’Malley and Nanson, 2002; Mattson et al., 2011; Kingdon et al., 2016). Moreover, although medication indicated specifically for ADHD that targets dopaminergic and noradrenergic signaling may reduce hyperactivity in children affected by FASD, it shows limited efficacy to mitigate attention deficits within this same population (Snyder et al., 1997; Oesterheld et al., 1998; Doig et al., 2008). To identify appropriate therapeutic strategies for affected children, it therefore is critical to determine the specific neurobiological mechanisms that underlie attention systems dysfunction in FASD (Paley and O’Connor, 2009; Peadon and Elliott, 2010; Koren, 2015).

Optimal attention performance depends on pyramidal neurons located within layer VI of the medial prefrontal cortex (mPFC). Approximately 40% of neurons within this population modulate the gain of corticothalamic signaling through projections to the mediodorsal thalamus via the thalamic reticular nucleus (Gabbott et al., 2005; Zikopoulos and Barbas, 2006; Olsen et al., 2012; Sherman, 2016), with the remaining 60% of neurons projecting to other targets including the hypothalamus, striatum, amygdala, and the prefrontal cortex itself (Gabbott et al., 2005; Hoover and Vertes, 2007). Layer VI neurons are stimulated directly by acetylcholine (ACh) activation of α4β2* type heteromeric nicotinic acetylcholine receptors (nAChRs), which are heteropentamers composed of two α4 subunits, two β2 subunits, and a fifth accessory subunit denoted by the asterisk that for mPFC layer VI neurons may be either an α4, β2, or α5 subunit (Kassam et al., 2008; Bailey et al., 2010, 2012; Poorthuis et al., 2013; Bloem et al., 2014). This action of ACh at mPFC layer VI pyramidal neurons contributes to the critical role of prefrontal cholinergic signaling to support optimal attention performance in situations requiring high attentional demand (Dalley et al., 2004; Parikh et al., 2007; Bailey et al., 2010; Howe et al., 2010; Guillem et al., 2011). Acute ethanol exposure increases ACh efficacy at α4β2* nAChRs (Aistrup et al., 1999; Cardoso et al., 1999; Zuo et al., 2004), whereas chronic ethanol exposure decreases α4β2* nAChR content (Robles and Sabriá, 2008; Hillmer et al., 2014) and may also decrease nAChR function *in vivo* (Majchrzak and Dilsaver, 1992). Chronic ethanol exposure during rat development impairs memory and attention in adulthood (Reyes et al., 1989; Nagahara and Handa, 1997; Woolfrey et al., 2005; Brys et al., 2014) and decreases the beneficial effects of nAChR stimulation to augment these mPFC-dependent functions (Nagahara and Handa, 1999). However, the long-term consequences of chronic developmental ethanol exposure to alter the function of mPFC layer VI pyramidal neurons, the function of nAChRs located on these neurons, and the ability of nicotinic signaling at these nAChRs to support attention behavior have not been determined. We find here that chronic developmental binge-pattern ethanol exposure in mice decreases performance on the five-choice serial reaction time test (5-CSRTT) for visual attention and dysregulates the function of mPFC layer VI pyramidal neurons, such that neurons show decreased intrinsic excitability along with increased responses to stimulation of both α4β2* nAChRs and α-amino-3-hydroxy-5-methyl-4-isoxazolepropionic acid (AMPA) glutamate receptors. Correlations between α4β2* nAChR function and performance on the 5-CSRTT are present in control mice but absent in mice exposed to ethanol during development, suggesting that this treatment disrupts the ability of nicotinic signaling in mPFC layer VI pyramidal neurons to support attention.

## Materials and Methods

### Experimental animals and breeding

C57BL/6 mice were purchased from Charles River Canada (Saint-Constant, QC, Canada) and bred in a secure vivarium at the University of Guelph. This facility had an ambient temperature of 21–24°C, and lights were maintained on a 12-h reverse light/dark cycle with lights on at 8:00 p.m. Nulliparous female mice aged 3–4 months were bred in pairs with male mice aged 4–5 months. Upon visual confirmation of a vaginal copulatory plug at the end of a dark cycle, female mice were separated to individual cages measuring 29 × 19 × 13 cm, and the next day was considered to be gestational day 1 (G1). All experimental animals in this study were cared for according to the principles and guidelines of the Canadian Council on Animal Care, and the experimental protocol was approved by the University of Guelph Animal Care Committee. Every effort was made to minimize animal suffering and limit the number of mice used in this study.

### Developmental treatment regimens

Pregnant female mice were randomly assigned to receive either ethanol or sucrose treatment via oral gavage from G10 to G18. Mice were administered ethanol at a dose of 2.0 g/kg/d [24.4% (w/v)] on G10 and G11, and 4.0 g/kg/d [48.9% (w/v)] from G12 to G18. Sucrose was administered in an amount that was isocaloric and isovolumetric to the ethanol treatment. Ethanol and sucrose solutions were made using tap water, and treatments were administered over two equally divided daily doses 2 h apart starting between 8:00 and 9:00 a.m. Mice in the ethanol treatment group received *ad libitum* access to water and pellet food (Tekland Global 18% Protein Rodent Maintenance Diet, Harlan Laboratories, Mississauga, ON, Canada). Mice in the sucrose treatment group received *ad libitum* access to water and were pair-fed with a mouse in the ethanol treatment group such that each mouse in the sucrose treatment group received the same amount of food as that eaten by its ethanol-treated pair for each day of gestation.

Pregnant mice and their litters were left undisturbed from G19 until postnatal day 4 (P4). The day of birth was considered to be P0. Individual pups were administered either ethanol or sucrose via oral gavage from P4 to P14 using a flexible plastic gavage needle (Instech Laboratories, Plymouth Meeting, PA). Postnatal treatment (ethanol or sucrose) was consistent with the prenatal treatment for each litter. Pups were administered ethanol at a dose of 1.5 g/kg/d [7.5% (w/v)] on P4 and P5 and 3.0 g/kg/d [15% (w/v)] from P6 to P14. Sucrose was administered in an amount that was isocaloric and isovolumetric to the ethanol treatment. Ethanol and sucrose solutions were prepared in Similac milk-based infant formula (Abbott Laboratories, Saint-Laurent, QC, Canada) using tap water. The milk formula within treatment solutions was prepared according to the manufacturer’s recommendations, except that the concentration was doubled on P4 and P5 to mitigate any decrease in nursing that may occur at the onset of postnatal treatment. Treatments were administered over two equally divided daily doses 2 h apart starting between 8:00 and 9:00 a.m. All mice in this study were weighed and monitored daily for general health during the breeding and treatment periods. Litters were weaned and separated based on sex on P28 into cages measuring 29 × 19 × 13 cm with a maximum of five mice per cage. Offspring were provided *ad libitum* access to water and pellet food (Tekland Global 16% Protein Rodent Maintenance Diet) and, with the exception of monitoring for general health and body weight, were left undisturbed until behavioral training began on P60.

### Blood ethanol concentration

Blood ethanol concentration (BEC) was measured for all dams on G15, which represents the midpoint for the 4 g/kg/d ethanol dosing regimen from G12 to G18. Ten microliters of blood was collected from the saphenous vein 1 h after the second daily gavage administration. BEC was measured in three naive litters not in this main study, which received ethanol treatment from P4 to P10. Pups were killed 1 h after the second daily gavage administration on P10 by decapitation under isoflurane anesthesia, and trunk blood was collected. P10 is the midpoint for the 3 g/kg/d ethanol dosing regimen for the pups from P6 to P14. For all analyses, 10 µl of blood was immediately added to 200 µl of 0.53N perchloric acid, mixed, and centrifuged at 14,000 × *g* for 15 min at 4°C. Supernatant (150 µl) was added to 150 µL of 0.53N potassium hydroxide, mixed, and stored at –80°C for later analysis. The concentration of short-chain alcohols in processed samples was measured using a microplate kit from Sigma-Aldrich Canada (Oakville, ON, Canada; product number MAK076) according to the manufacturer’s recommendations.

### Five-choice serial reaction time test

The 5-CSRTT was performed using Bussey–Saksida mouse touch screen–operant conditioning chambers (Lafayette Instrument Co., Lafayette, IN). Trapezoid-shaped chambers with 188-cm^2^ floor space housed a perforated stainless steel floor and a thin-film transistor touchscreen display on one wall. A plastic mask was fixed over the touchscreen that contained five square cut-outs measuring 4 × 4 cm, which created five distinct areas for light stimulus presentation and nose poke touch response. The opposite wall contained a reinforcer magazine equipped with a photodetector, light, and reward trough, where 7 μl of Neilson strawberry milkshake (Saputo Dairy Products Canada G.P., Saint-Laurent, QC, Canada) could be delivered by a peristaltic pump. Chambers were controlled by a personal computer running a 5-CSRTT application on the ABET II interface software (model 89543, Lafayette Instrument) and were housed in sound-attenuating cubicles equipped with a ventilation fan.

Starting at P60, 16 male mice from nine ethanol-treated litters and 16 male mice from eight sucrose-treated litters were pair-housed within cages measuring 29 × 19 × 13 cm with *ad libitum* access to water. Mice were randomly sampled as 1–3 mice per litter in the ethanol treatment group and 1–4 mice per litter in the sucrose treatment group. For the measures in this study that were significantly affected by developmental treatment, one-way ANOVA followed by Dunnett’s *post hoc* test confirmed that the mean for no single litter was significantly different from the mean of its treatment group. Mice were food restricted to maintain a body weight of approximately 85% of their free-feeding body weight. Training on the 5-CSRTT was performed according to the 89543CAM 5-Choice Serial Reaction Time Task with Cambridge Amendment Manual (Lafayette Instrument) with minor alterations. Behavioral testing was performed 6 d per week (Sunday to Friday) and occurred at a similar time of day for each mouse between 9:00 a.m. and 3:00 p.m., which corresponded with the dark cycle for this study. The house light remained off for all sessions and was illuminated only during timeout periods. Training began with sessions of habituation to the chamber and reward delivery, which throughout this study was accompanied by the illumination of the magazine light and the emission of a short tone (3 KHz for 1 s). This was followed by one session of Pavlovian conditioning to link stimulus presentation with reward delivery. Daily touch-response training sessions began with a mouse placed in a chamber with one of five stimulus locations illuminated. A nose poke response in that stimulus location extinguished its light and resulted in reward delivery. Entrance into the magazine extinguished the magazine light and initiated a 5-s intertrial interval (ITI) to the next stimulus presentation. Stimuli were presented in a pseudorandom order, and mice were required to complete 30 trials within 60 min on two consecutive days to proceed. Daily training sessions for trial initiation built on the previous scheme, with the modification that the magazine light illuminated at the end of the ITI and a nose poke into the magazine was required to extinguish its light and start a 5-s delay to the next stimulus presentation. Mice were required to complete 30 trials within 60 min on two consecutive days to proceed.

Training sessions for the complete 5-CSRTT protocol began with a mouse placed in the chamber with the magazine light illuminated. A nose poke into the magazine extinguished its light and started the first trial with a 5-s delay to one of the five stimulus locations illuminating for a brief period. A nose poke response in that stimulus location while it was illuminated or during the following 5-s limited hold period resulted in reward delivery. Entrance into the magazine to collect the reward started a 5-s ITI, after which the magazine light illuminated and the mouse was required to re-enter the magazine to extinguish its light and start the next trial. A premature response made between trial initiation and stimulus presentation was not rewarded and led to a 5-s timeout period with the house light illuminated followed by a 5-s ITI, after which that same trial could be reinitiated by a nose poke into the magazine. An incorrect response in one of the four stimulus locations that was not illuminated or an error of omission in which no response was made by the end of the limited hold period was not rewarded and led to a 5-s timeout period with the house light illuminated, followed by a 5-s ITI, after which a magazine response initiated the next trial. Daily sessions lasted for 60 trials or 60 min, and each stimulus location was presented 12 times in a pseudorandom order. Accuracy percentage was calculated as [number of correct responses/(number of correct responses + number of incorrect responses) × 100]. Percentage of omissions was calculated as (omissions/total number of trials) × 100. Training began with an initial stimulus duration of 8 s, and this was gradually reduced depending on performance to a final stimulus duration of 1 s. The criterion to advance to the next stimulus duration was a performance of 60 trials with >80% accuracy and <20% omissions for three of four consecutive sessions.

### Brain slice preparation for electrophysiology

Mice were left undisturbed with *ad libitum* access to food and water for approximately 2 weeks after the completion of behavioral testing. Mice were killed by decapitation under isoflurane anesthesia, and brains were removed rapidly and cooled for 2 min in 4°C oxygenated sucrose artificial cerebral spinal fluid (ACSF; 254 mm sucrose, 10 mm d-glucose, 26 mm NaHCO_3_, 2 mm CaCl_2_, 2 mm MgSO_4_, 3 mm KCl, and 1.25 mm NaH_2_PO_4_, pH 7.4). Coronal slices containing the mPFC were cut in 4°C oxygenated sucrose ACSF at 400-μm thickness using a Leica VT 1200 vibrating microtome (Leica Microsystems, Concord, ON, Canada). The appearance of white matter and the corpus callosum were used as anterior and posterior landmarks (Paxinos and Franklin, 2001; Gabbott et al., 2005). Slices were placed in a recovery chamber (Scientific Systems Design, Mississauga, ON, Canada) with 30°C oxygenated ACSF (128 mm NaCl, 10 mm d-glucose, 26 mm NaHCO_3_, 2 mm CaCl_2_, 2 mm MgSO_4_, 3 mm KCl, 1.25 mm NaH_2_PO_4_, pH 7.4) for at least 2 h before the beginning of electrophysiological recording.

### Electrophysiology

Brain slices were transferred to a modified recording chamber (Warner Instruments, Hamden, CT) mounted onto the stage of an Axioskop FS2 microscope (Carl Zeiss Canada, Toronto, ON, Canada) and superfused with oxygenated room-temperature ACSF at a rate of 3–4 ml/min. Pyramidal neurons in layer VI were visualized using infrared differential interference contrast microscopy and identified based on location within seven cell bodies (approximately 150 μm) from the medial aspect of the white matter and also by the presence of a prominent apical dendrite (Bailey et al. 2012; Tian et al. 2014). Neurons were sampled from the anterior cingulate, prelimbic, and infralimbic cortical regions, and there was no effect of sampling location on any measure in this study. Whole-cell recording was performed using borosilicate glass pipette electrodes (2–5 MΩ; Sutter Instrument Company, Novato, CA) containing 120 mm K-gluconate, 5 mm KCl, 2 mm MgCl_2_, 4 mm K_2_-ATP, 400 μM Na_2_-GTP, 10 mm Na_2_-phosphocreatine, and 10 mm HEPES buffer (adjusted to pH 7.3 with KOH). Recordings were made using a Multiclamp 700B amplifier, acquired at 20 kHz, low-pass filtered at 2 kHz using a Digidata 1440A data acquisition system (Molecular Devices, Sunnyvale, CA), and corrected for the liquid junction potential. Neuron passive and active electrophysiological properties were first determined in current-clamp mode by measuring changes to membrane potential from rest in response to positive and negative current steps. Burst-firing neurons and fast-spiking interneurons were not used for subsequent analyses because they respond primarily to indirect nicotinic stimulation (Kassam et al. 2008).

Neurons were next held at –75 mV in voltage-clamp mode for 5 min to record their baseline spontaneous excitatory postsynaptic currents (sEPSCs). Neurons remained at –75 mV, and receptor-mediated inward current responses were measured as follows: Nicotinic responses were probed by the addition of 1 mm ACh (Sigma-Aldrich Canada) after a minimum 10-min pre-exposure to 200 nM atropine; muscarinic responses were probed by the addition of 1 mm ACh after a minimum 10-min pre-exposure to 3 μM dihydro-β-erythroidine hydrobromide (DHβE; Tocris Bioscience/Bio-Techne, Minneapolis, MN); and AMPA glutamatergic responses were probed by the addition of 2 μM (S)-AMPA (Tocris Bioscience). All agonists were applied in the bath for 15 s. In mPFC layer VI pyramidal neurons, the nicotinic response to bath application of ACh is inhibited by the α4β2* nAChR antagonist DHβE but not by the α7 nAChR antagonist methyllycaconitine (Kassam et al., 2008; Bailey et al., 2010; Poorthuis et al., 2013), suggesting that all nicotinic responses in this study were mediated by α4β2* nAChRs. Current responses were measured using Clampfit 10.3 software (Molecular Devices) as the change in holding current from baseline to the peak of the response. Receptor-mediated acceleration of action potential firing was measured in current-clamp mode by first injecting sufficient positive current to produce an approximate 1-Hz baseline firing frequency. After a minimum 30 s of stable baseline, each agonist was applied in the bath as described above. The percentage increase in firing frequency in response to agonist application was measured for each neuron as [(frequency at the peak of the drug response – frequency at baseline)/frequency at baseline] × 100.

### Statistical analysis

BEC, pregnancy outcome, and offspring body weight data are presented as dam/litter mean ± 1 SEM of eight to nine litters for each treatment group, with the litter as the unit of determination for statistical analyses. Behavioral data on the 5-CSRTT are presented as mean ± 1 SEM of 14–16 male offspring from eight to nine litters for each treatment group, and electrophysiological data are presented as mean ± 1 SEM for 12 to 114 neurons from the same mice that were tested on the 5-CSRTT task. The unit of determination for statistical analyses was the mouse for behavioral experiments and the neuron for electrophysiology experiments. Data sets were first analyzed for normality and homogeneity of variance before statistical comparisons were performed. The statistical test used for each comparison is indicated in Results, tables, and figure legends, and all statistical tests along with their results are compiled in Table 1. These included the two-tailed unpaired *t* test (for normally distributed data sets), the Mann–Whitney *U* test (for non–normally distributed data sets), and the two-way repeated-measures ANOVA followed by the Bonferroni *post hoc* test. The Pearson correlation coefficient was used to assess relationships between electrophysiological measures and attention performance on the 5-CSRTT. Statistical analyses were performed using GraphPad Prism 6 (GraphPad Software, La Jolla, CA).

**Table 1.** Statistics.

Line	Location	Type of test	*p*-value
a	Table 2	Two-tailed Mann-Whitney *U* test (gestation length)	0.3
b	Table 2	Two-tailed unpaired *t* test (litter size)	0.9
c	Table 2	Bonferroni’s *post hoc* test (body weight at P4)	1.0
d	Table 2	Bonferroni’s *post hoc* test (body weight at P14)	1.0
e	Table 2	Bonferroni’s *post hoc* test (body weight at P21)	1.0
f	Table 2	Bonferroni’s *post hoc* test (body weight at P28)	1.0
g	Table 2	Bonferroni’s *post hoc* test (body weight at P60)	1.0
h	Fig. 2A	Two-way repeated-measures ANOVA (stimulus duration)	*F*_(7,203)_ = 13.2; *p* < 0.0001
i	Fig. 2A	Two-way repeated-measures ANOVA (treatment)	*F*_(1,29)_ = 6.9; *p* = 0.01
j	Fig. 2A	Bonferroni’s *post hoc* test at 8 s	*p* = 0.04
k	Fig. 2A	Bonferroni’s *post hoc* test at 1 s	*p* = 0.0001
l	Fig. 2B	Two-way repeated-measures ANOVA (stimulus duration)	*F*_(7,203)_ = 178.2; *p* < 0.0001
m	Fig. 2B	Two-way repeated-measures ANOVA (treatment)	*F*_(1,29)_ = 2.3; *p* = 0.1
n	Fig. 2B	Two-way repeated-measures ANOVA (stimulus duration X treatment)	*F*_(7,203)_ = 5.3; *p* < 0.0001
o	Fig. 2B	Bonferroni’s *post hoc* test at 8 s	*p* <0.0001
p	Fig. 2C	Two-way repeated-measures ANOVA (stimulus duration)	*F*_(7,203)_ = 26.7, *p* < 0.0001
q	Fig. 2C	Two-way repeated-measures ANOVA (treatment)	*F*_(1,29)_ = 0.2; *p* = 0.6
r	Fig. 2C	Two-way repeated-measures ANOVA (stimulus duration × treatment)	*F*_(7,203)_ = 2.8; *p* = 0.009
s	Fig. 2C	Bonferroni’s *post hoc* test at 8 s	*p* = 0.005
t	Fig. 2D	Two-way repeated-measures ANOVA (stimulus duration)	*F*_(7,203)_ = 38.4; *p* < 0.0001
u	Fig. 2D	Two-way repeated-measures ANOVA (treatment)	*F*_(1,29)_ = 7.1; *p* = 0.01
v	Fig. 2D	Two-way repeated-measures ANOVA (stimulus duration × treatment)	*F*_(7,203)_ = 3.2; *p* = 0.003
w	Fig. 2D	Bonferroni’s *post hoc* test at 1.2 s	*p* = 0.04
x	Fig. 2D	Bonferroni’s *post hoc* test at 1.0 s	*p* < 0.0001
y	Fig. 2E	Two-way repeated-measures ANOVA (stimulus duration)	*F*_(7,203)_ = 58.6; *p* < 0.0001
z	Fig. 2E	Two-way repeated-measures ANOVA (treatment)	*F*_(1,29)_ = 0.8; *p* = 0.4
aa	Fig. 2F	Two-way repeated-measures ANOVA (stimulus duration)	*F*_(7,203)_ = 430.1; *p* < 0.0001
ab	Fig. 2F	Two-way repeated-measures ANOVA (treatment)	*F*_(1,29)_ = 0.01; *p* = 0.9
ac	Fig. 3A	Two-way repeated-measures ANOVA (stimulus duration)	*F*_(7,203)_ = 73.1; *p* < 0.0001
ad	Fig. 3A	Two-way repeated-measures ANOVA (treatment)	*F*_(1,29)_ = 1.3; *p* = 0.3
ae	Fig. 3A	Two-way repeated-measures ANOVA (stimulus duration × treatment)	*F*_(7,203)_ = 2.5; *p* = 0.02)
af	Fig. 3A	Bonferroni’s *post hoc* test at 8 s	*p* = 0.001
ag	Fig. 3B	Two-way repeated-measures ANOVA (stimulus duration)	*F*_(7,203)_ = 42.8; *p* < 0.0001
ah	Fig. 3B	Two-way repeated-measures ANOVA (treatment)	*F*_(1,29)_ = 5.6; *p* = 0.03
ai	Fig. 3B	Bonferroni’s *post hoc* test at 1.6 s	*p* = 0.02
aj	Fig. 3B	Bonferroni’s *post hoc* test at 1.0 s	*p* = 0.04
ak	Table 3	Two-tailed Mann-Whitney *U* test (capacitance)	*p* = 0.002
al	Table 3	Two-tailed Mann-Whitney *U* test (input resistance)	*p* = 0.09
am	Table 3	Two-tailed Mann-Whitney *U* test (resting membrane potential)	*p* = 0.5
an	Table 3	Two-tailed Mann-Whitney *U* test (spike amplitude)	*p* = 0.8
ao	Fig. 4A	Two-tailed unpaired *t* test	*p* = 0.009
ap	Fig. 4B	Two-way repeated-measures ANOVA (current injected × treatment)	*F*_(10,1930)_ = 4.7; *p* < 0.0001
aq	Fig. 4B (rising phase)	Two-way repeated-measures ANOVA (current injected)	*F*_(3,579)_ = 922.1; *p* < 0.0001
ar	Fig. 4B (rising phase)	Two-way repeated-measures ANOVA (treatment)	*F*_(1,193)_ = 4.9; *p* = 0.03
as	Fig. 4B (rising phase)	Two-way repeated-measures ANOVA (current injected × treatment)	*F*_(3,579)_ = 2.3; *p* = 0.07
at	Fig. 4B (descending phase)	Two-way repeated-measures ANOVA (current injected)	*F*_(3,579)_ = 144.3; *p* < 0.0001
au	Fig. 4B (descending phase)	Two-way repeated-measures ANOVA (treatment)	*F*_(1,193)_ = 4.2, *p* = 0.04
av	Fig. 4B (descending phase)	Two-way repeated-measures ANOVA (current injected × treatment)	*F*_(3,579)_ = 0.3; *p* = 0.8
aw	Fig. 4D	Two-way repeated-measures ANOVA on log-transformed data (current injected)	*F*_(1,174)_ = 56.4; *p* < 0.0001
ax	Fig. 4D	Two-way repeated-measures ANOVA on log-transformed data (treatment)	*F*_(1,174)_ = 5.2; *p* = 0.02
ay	Fig. 4D	Two-way repeated-measures ANOVA on log-transformed data (current injected × treatment)	*F*_(1,174)_ = 0.04; *p* = 0.8
az	Fig. 4D	Two-tailed Mann-Whitney *U* test (at 100 pA)	*p* = 0.03
ba	Fig. 4D	Two-tailed Mann-Whitney *U* test (at 250 pA)	*p* = 0.04
bb	Fig. 5A	Two-tailed unpaired *t* test	*p* = 0.01
bc	Fig. 5B	Two-tailed Mann-Whitney *U* test	*p* = 0.008
bd	Fig. 5C1	Two-way repeated-measures ANOVA (time)	*F*_(11,1419)_ = 30.5; *p* < 0.0001
be	Fig. 5C1	Two-way repeated-measures ANOVA (treatment)	*F*_(1,1419)_ = 35.8; *p* < 0.0001
bf	Fig. 5C2	Two-tailed unpaired *t* test	*p* = 0.01
bg	Fig. 5C3	Two-tailed Mann-Whitney *U* test	*p* = 0.01
bh	Table 4	Two-tailed Mann-Whitney *U* test (frequency)	*p* = 0.6
bi	Table 4	Two-tailed Mann-Whitney *U* test (amplitude)	*p* = 0.08
bj	Table 4	Two-tailed Mann-Whitney *U* test (10–90 rise)	*p* = 0.0008
bk	Table 4	Two-tailed Mann-Whitney *U* test (10–90 slope)	*p* = 0.02
bl	Table 4	Two-tailed Mann-Whitney *U* test (decay)	*p* = 0.9
bm	Fig. 7A	Two-tailed Mann-Whitney *U* test	*p* = 0.1
bn	Fig. 7B	Two-tailed unpaired *t* test	*p* = 0.04
bo	Fig. 7C1	Two-way repeated-measures ANOVA (time)	*F_(11,311)_ = 4.0*; *p < 0.0001*
bp	Fig. 7C1	Two-way repeated-measures ANOVA (treatment)	*F_(1,311)_ = 5.4*; *p = 0.02*
bq	Fig. 7C2	Two-tailed unpaired *t* test	*p* = 0.6
br	Fig. 7C3	Two-tailed unpaired *t* test	*p* = 0.047
bs	Table 5	Two-tailed Pearson correlation coefficient	As indicated
bt	Table 6	Two-tailed Pearson correlation coefficient	As indicated

## Results

**Figure 1.**
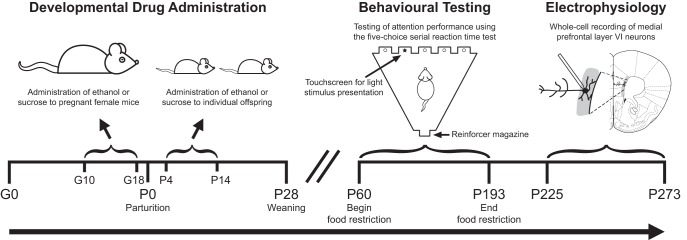
Schematic illustration of the study design. Timed-pregnant female mice were administered either ethanol or an isocaloric/isovolumetric amount of sucrose by gavage from G10 to G18. Offspring were then administered the same treatment (ethanol or sucrose) by gavage from P4 to P14. Male offspring were food-restricted and tested for attention behavior using the 5-CSRTT from P60 to P193 (the age of the oldest mouse to complete testing). The same cohort of male offspring was then tested for electrophysiological function of medial prefrontal layer VI pyramidal neurons between P225 and P273. The coronal slice diagram was modified from Paxinos and Franklin, 2001. Timelines are not drawn to scale.

The objective of this study was to determine long-term consequences of developmental binge-pattern ethanol exposure on performance in an attention task and on the function of mPFC layer VI pyramidal neurons that support attention processing. Developing mice were administered ethanol or isocaloric/isovolumetric sucrose control from G10 to G18 and P4 to P14. Attention performance was measured in adulthood using the 5-CSRTT, and the function of mPFC layer VI neurons from these same mice was assessed using whole-cell electrophysiology in acute brain slices. Refer to Figure 1 for a schematic of the study design. BEC of pregnant mice 1 h after the second daily administration of ethanol on G15 was 234.8 ± 34.2 mg/dl (*n* = 9). The BEC of mice from three separate litters that were administered ethanol from P4 to P10 (and not included in the remainder of this study) 1 h after the second daily administration of ethanol on P10 was 255.2 ± 43 mg/dl (*n* = 14). These BEC values are similar to those found in previous studies following binge-pattern oral administration of ethanol to developing mice (Jiang et al., 2007; Cui et al., 2010; Kane et al., 2011), rats (Maier et al., 1996; Ryan et al., 2008; Brocardo et al., 2012) and guinea pigs (Bailey et al., 2001; Iqbal et al., 2006; Olmstead et al., 2009), where neurocognitive and neurological teratogenic effects were observed. It should be noted that these BEC values are also similar to those predicted in a recent ethanol pharmacokinetic modeling study for pregnant mice after a single 4-g/kg oral dose of ethanol (Martin et al., 2015). However, the same study found these values to be approximately one-half of those predicted for pregnant humans after the same ethanol dose (Martin et al., 2015). There was no effect of ethanol treatment on the amount of food consumed by dams or litters, although there was a small decrease in the amount of food consumed by dams of both groups on the first day of treatment only (data not shown). We observed no effect of treatment on the length of gestation, litter size at P4, or offspring body weight at any point during postnatal development (all reported in Table 2).

**Table 2.** Pregnancy outcome and offspring body weight.

Characteristic	Sucrose	Ethanol	*p*-value
Number of litters	8	9	
Gestation length (d)	19.9 ± 0.1	20.3 ± 0.2	0.3^a^
Litter size (number of pups at P4)	8.4 ± 0.9	8.2 ± 0.6	0.9^b^
Offspring body weight (g)			
P4 (female and male)	2.8 ± 0.1	2.9 ± 0.1	1.0^c^
P14 (female and male)	7.1 ± 0.2	6.9 ± 0.1	1.0^c^
P21 (male only)	9.8 ± 0.6	9.6 ± 0.2	1.0^c^
P28 (male only)	16.5 ± 0.9	16.7 ± 0.3	1.0^c^
P60 (male only)	24.6 ± 0.8	24.7 ± 0.2	1.0^c^

Data are presented as litter mean ± 1 SEM. Data sets were analyzed by ^a^Mann–Whitney *U* test, ^b^two-tailed unpaired *t* test, or ^c^Bonferroni’s *post hoc* test.

### Developmental ethanol exposure impairs performance on an attention task in adulthood

We first sought to measure performance of adult offspring on the 5-CSRTT (Robbins, 2002), because deficits in attention make up one of the most common and persistent neurobehavioral consequences of prenatal ethanol exposure in humans (Bhatara et al., 2006; Fryer et al., 2007). Thirty-two young adult male mice (*n* = 16 for each developmental treatment group sampled randomly from eight sucrose-treated litters and nine ethanol-treated litters) were trained to detect and respond to an illuminating light stimulus presented randomly in one of five locations, to receive a reinforcing food reward. Training on the 5-CSRTT began with a stimulus duration of 8 s, and this was decreased in successive steps to increase attentional demand until the final stimulus duration of 1 s was reached. Mice were required to meet the criteria of 60 trials completed within 60 min with >80% accuracy and <20% omissions on three of four consecutive daily sessions to advance to the next stimulus duration. A full description of the training procedure is presented in Materials and Methods. One mouse in the sucrose treatment group stopped performing the task during this behavioral testing and was removed from all analyses.

**Figure 2.**
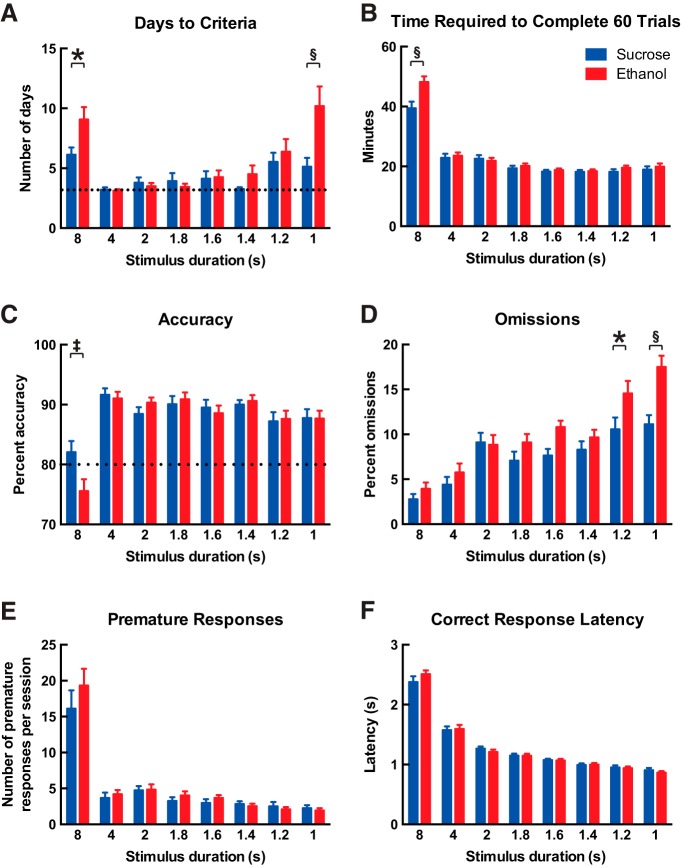
Developmental ethanol exposure impairs performance on an attention task in adulthood. Adult male offspring were trained on the 5-CSRTT for visual attention. Training began with the light stimulus duration set to 8 s, and each mouse was required to achieve the criteria of (i) 60 trials completed in 60 min, (ii) >80% accuracy, and (iii) <20% omissions for three of four consecutive days to advance to the next-lowest stimulus duration. The number of days required to meet criteria at each stimulus duration is shown in ***A***, where the dotted line represents the minimum of 3 days. Mice that were administered ethanol during development required more days to reach criteria than mice that were administered sucrose during development, both during initial training on the task and also at the lowest stimulus duration that required the highest attentional demand (two-way repeated-measures ANOVA, effect of developmental treatment, *p* = 0.01; effect of stimulus duration, *p* < 0.0001; interaction, *p* = 0.001; Bonferroni’s *post hoc* test at 8 s, *p = 0.04, and at 1 s, ^§^*p* < 0.0001). All remaining data are shown as the mean performance for all days up to and including the day on which each mouse met training criteria for each stimulus duration. ***B***, Mice that were administered ethanol during development required more time to complete 60 trials at the initial 8-s stimulus duration (effect of developmental treatment, *p* = 0.1; effect of stimulus duration, *p* < 0.0001; interaction, *p* < 0.0001; Bonferroni’s *post hoc* test at 8 s, ^§^*p* < 0.0001). Mice that were administered ethanol showed lower accuracy at the initial 8-s stimulus duration (***C***, effect of developmental treatment, *p* = 0.6; effect of stimulus duration, *p* < 0.0001; interaction, *p* = 0.009; Bonferroni’s *post hoc* test at 8 s, ^‡^*p* = 0.005), and also showed greater omissions, which was most pronounced at lower stimulus durations (***D***, effect of developmental treatment, *p* = 0.01; effect of stimulus duration, *p* < 0.0001; interaction, *p* = 0.003; Bonferroni’s *post hoc* test at 1.2 s, *p = 0.04, and at 1 s, ^§^*p* < 0.0001). ***E***, The number of premature responses per session was affected by stimulus duration (*p* < 0.0001) but not by developmental treatment (*p* = 0.4). ***F***, The latency to make correct responses also was affected by stimulus duration (*p* < 0.0001) but not by developmental treatment (*p* = 0.9). All data are shown as mean + 1 SEM.

The number of days (sessions) required to reach criteria at each stimulus duration was significantly affected by stimulus duration, where mice required the greatest number of days both during initial training on the task (8 s) and also at the shorter stimulus durations (1.2 and 1.0 s) that involve greater attentional demand (Fig. 2*A*, two-way repeated-measures ANOVA, effect of stimulus duration, *F*_(7,203)_ = 13.2; *p* < 0.0001). Mice from the ethanol treatment group required more days to reach criteria than mice in the sucrose treatment group (effect of developmental treatment, *F*_(1,29)_ = 6.9; *p* = 0.01), and this effect of treatment was most pronounced at both the initial 8 s (Bonferroni’s *post hoc* test, *p* = 0.04) and the shortest 1 s (*p* <0.0001) stimulus durations. The remainder of data in Fig. 2 are presented as means for all days up to and including the day when each mouse met criteria for each stimulus duration. Mice in the ethanol treatment group required more time to complete 60 trials than mice in the sucrose treatment group at 8 s only (Fig. 2*B*; effect of stimulus duration, *F*_(7,203)_ = 178.2; *p* < 0.0001; effect of developmental treatment, *F*_(1,29)_ = 2.3; *p* = 0.1; effect of interaction, *F*_(7,203)_ = 5.3; *p* < 0.0001, Bonferroni’s *post hoc* test at 8 s, *p* <0.0001). Mice responded with the lowest accuracy percentage at 8 s (Fig. 2*C*; effect of stimulus duration, *F*_(7,203)_ = 26.7, *p* < 0.0001), whereas mice in the ethanol treatment group showed a lower percent accuracy than mice in the sucrose treatment group (main effect of developmental treatment, *F*_(1,29)_ = 0.2; *p* = 0.6; effect of interaction, *F*_(7,203)_ = 2.8; *p* = 0.009, Bonferroni’s *post hoc* test at 8 s, *p* = 0.005). As shown in Figure 2*D*, the percentage of omissions (no response) increased as stimulus duration decreased (effect of stimulus duration, *F*_(7,203)_ = 38.4; *p* < 0.0001), and this effect was most pronounced in mice from the ethanol treatment group, as they showed greater percentages of omissions than mice in the sucrose treatment group at 1.2 and 1.0 s (effect of developmental treatment, *F*_(1,29)_ = 7.1; *p* = 0.01; effect of interaction, *F*_(7,203)_ = 3.2; *p* = 0.003, Bonferroni’s *post hoc* test at 1.2 s, *p* = 0.04, and at 1.0 s, *p* < 0.0001). The number of premature responses per session was greatest at 8 s (Fig. 2*E*; effect of stimulus duration, *F*_(7,203)_ = 58.6; *p* < 0.0001) but was not affected by treatment (*F*_(1,29)_ = 0.8; *p* = 0.4). Similarly, as shown in Figure 2*F*, correct response latency was affected by stimulus duration (*F*_(7,203)_ = 430.1; *p* < 0.0001) but not by treatment (*F*_(1,29)_ = 0.01; *p* = 0.9). The number of responses per session that were perseverative to the correct response was not affected by stimulus duration (data not shown; *F*_(7,203)_ = 1.1; *p* = 0.3) or developmental treatment (*F*_(1,29)_ = 2.5; *p* = 0.1). Reward collection latency was greatest at 8 s compared with the other stimulus durations (data not shown; effect of stimulus duration, *F*_(7,203)_ = 12.7; *p* < 0.0001) but was not affected by developmental treatment (*F*_(1,29)_ = 0.03; *p* = 0.9).

**Figure 3.**
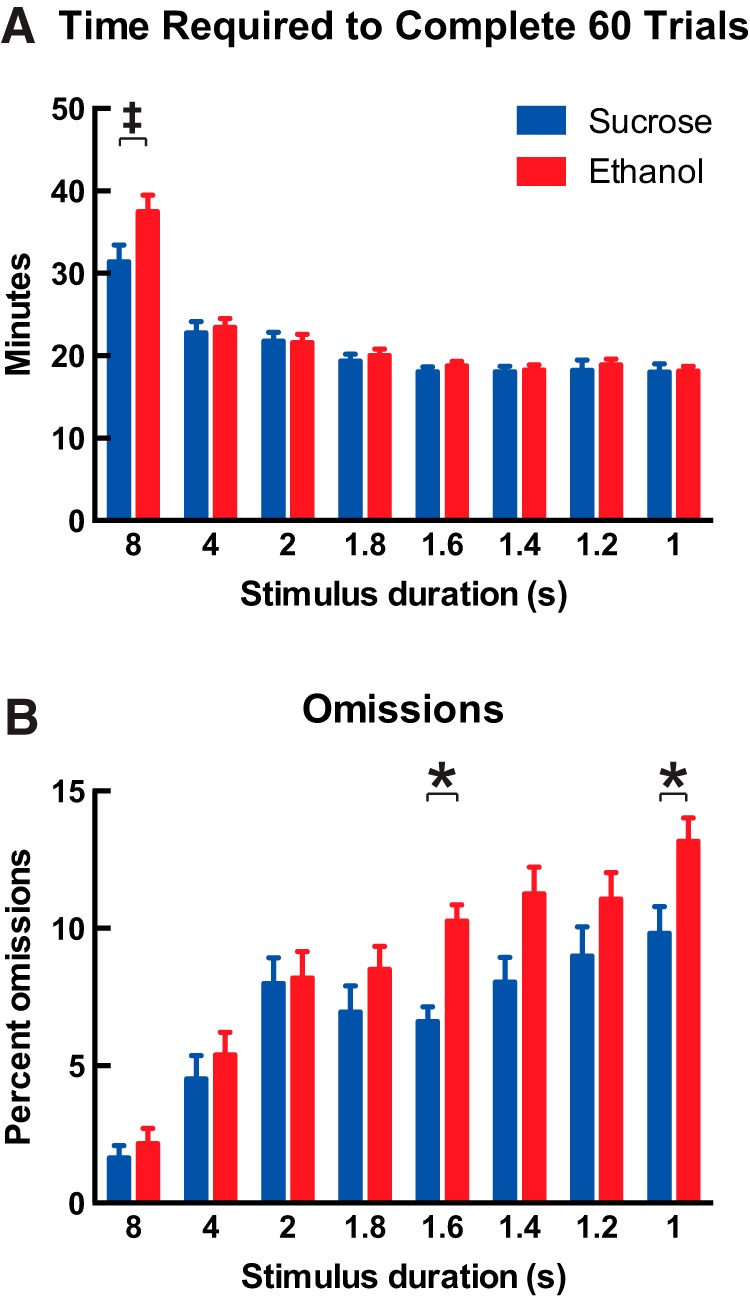
Developmental ethanol exposure impairs performance on the 5-CSRTT even when mice are considered to be trained. Data are shown as means for the 3 days on which each mouse met the training criteria for each stimulus duration. ***A***, Mice that were administered ethanol during development required more time to complete 60 trials at the 8-s stimulus duration than mice that were administered sucrose during development (two-way repeated-measures ANOVA; effect of developmental treatment, *p* = 0.3; effect of stimulus duration, *p* < 0.0001; interaction, *p* = 0.01; Bonferroni’s *post hoc* test at 8 s, ^‡^*p* = 0.001). ***B***, Mice that were administered ethanol during development committed more errors of omission when trained on the task, and this effect was most prominent at lower stimulus durations that required higher attentional demand (effect of developmental treatment, *p* = 0.03; effect of stimulus duration, *p* < 0.0001; interaction, *p* = 0.04; Bonferroni’s *post hoc* test at 1.6 s, *p = 0.02, and at 1 s, *p = 0.04). All data are shown as mean + 1 SEM.

Mice from the ethanol treatment group continued to show impaired performance on the 5-CSRTT even when they were considered to be fully trained. We next analyzed data only for the 3 days on which each mouse met training criteria for each stimulus duration. The time required to complete 60 trials was affected by stimulus duration (Fig. 3*A*; two-way repeated-measures ANOVA, *F*_(7,203)_ = 73.1; *p* < 0.0001), and although there was no main effect of developmental treatment (*F*_(1,29)_ = 1.3; *p* = 0.3), there was a significant interaction between effects (*F*_(7,203)_ = 2.5; *p* = 0.02) and mice from the ethanol treatment group requiring more time to complete 60 trials at 8 s than mice in the sucrose treatment group (Bonferroni’s *post hoc* test, *p* = 0.001). As shown in Figure 3*B* for the percentage of omissions, it is most interesting that effects of stimulus duration (*F*_(7,203)_ = 42.8; *p* < 0.0001) and developmental treatment (*F*_(1,29)_ = 5.6; *p* = 0.03, Bonferroni’s *post hoc* test at 1.6 s, *p* = 0.02, and at 1.0 s, *p* = 0.04) persisted in mice that were fully trained on the task. The same analysis for the other measures within the 5-CSRTT did not show effects of developmental treatment in the trained mice (data not shown). Accuracy (*F*_(7,203)_ = 3.7; *p* = 0009), premature responding (*F*_(7, 203)_ = 22.0; *p* < 0.0001), correct response latency (*F*_(7, 203)_ = 250.2; *p* < 0.0001), and reward collection latency (*F*_(7, 203)_ = 8.1; *p* < 0.0001) were all affected by stimulus duration but not by developmental treatment (all *p* > 0.05). The number of responses that were perseverative to the correct response was not affected by stimulus duration or developmental treatment (both *p* > 0.05).

### Developmental ethanol exposure decreases the intrinsic excitability of prefrontal layer VI pyramidal neurons

We next sought to determine whether developmental binge-pattern ethanol exposure influences the function of adult mPFC layer VI pyramidal neurons, because approximately 40% of neurons in this population provide feedback from the mPFC to the thalamus (Gabbott et al., 2005; Zikopoulos and Barbas, 2006) and ACh neurotransmission via their α4β2* nAChRs is necessary for proper attention performance (Bailey et al., 2010; Guillem et al., 2011). We prepared acute brain slices from the same mice that had been tested on the 5-CSRTT and first measured the basic passive and active electrophysiological properties of layer VI neurons. The brain from one mouse in the sucrose treatment group was lost to a technical issue, leaving 14 mice in the sucrose treatment group and 16 mice in the ethanol treatment group for experiments. Data and statistical analysis of basic electrophysiological properties are shown in Table 3. Neurons from mice in the ethanol treatment group showed significantly lower capacitance (Mann–Whitney *U* test, *p* = 0.002) and a trend toward higher input resistance (*p* = 0.09) compared with neurons from mice in the sucrose treatment group. There was no effect of developmental treatment on resting membrane potential or spike amplitude (both *p* > 0.05).

**Table 3.** Basic electrophysiological properties of prefrontal layer VI pyramidal neurons.

Characteristic	Sucrose	Ethanol	*p*-value
Number of mice	14	16	
Number of neurons	104	114	
Capacitance (pF)	56.9 ± 0.9	53.4 ± 0.9	0.002*
Input resistance (MΩ)	228.7 ± 7.5	240.6 ± 7.8	0.09
Resting membrane potential (mV)	–78.7 ± 0.5	–78.3 ± 0.4	0.5
Spike amplitude (mV)	95.0 ± 0.5	94.7 ± 0.5	0.8

Data are presented as mean ± 1 SEM for neurons within each data set. Data sets were analyzed by Mann–Whitney *U* test. *Statistically significant (*p* < 0.05).

**Figure 4.**
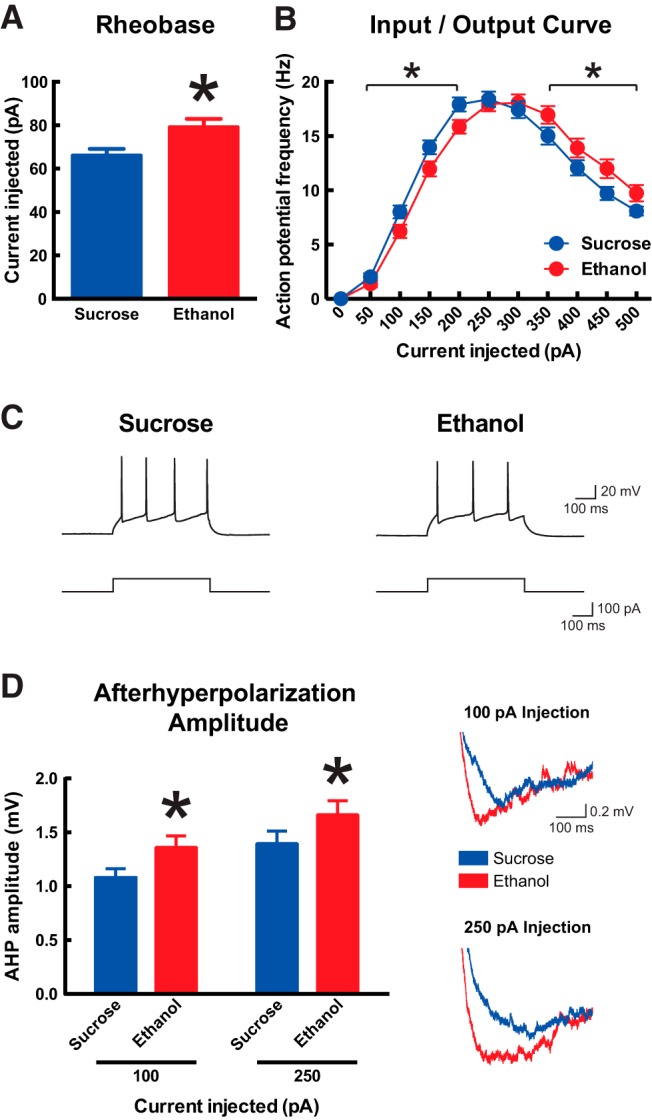
Developmental ethanol exposure decreases the intrinsic excitability of adult medial prefrontal layer VI pyramidal neurons. ***A***, Neurons from mice that were administered ethanol during development required more current to reach action potential threshold from rest (rheobase) than neurons from mice that were administered sucrose during development (two-tailed unpaired *t* test, *p = 0.009). ***B***, The input–output curve is shifted to the right in neurons from mice that were administered ethanol during development (two-way repeated-measures ANOVA; interaction between effects of current and developmental treatment, *p* < 0.0001; effect of developmental treatment within each indicated segment, *p < 0.04). Representative action potential trains elicited by 100-pA current steps are shown in ***C*** for one neuron from each developmental treatment group. ***D***, AHP amplitude at the end of the action potential trains elicited by 100- and 250-pA current steps is greater in neurons from mice that were administered ethanol during development (two-way repeated-measures ANOVA on log-transformed data, *p* = 0.02; Mann–Whitney *U* test on raw data for each current step, *p* < 0.04). Representative AHP traces are shown on the right for one neuron from each developmental treatment group. All data are shown as mean ± 1 SEM.

Measures of intrinsic excitability for mPFC layer VI pyramidal neurons are shown in Figure 4. The amount of positive current injection required to reach action potential threshold from rest (rheobase) was significantly greater in neurons from mice in the ethanol treatment group (78.9 ± 3.9 pA, *n* = 90) than in neurons from mice in the sucrose treatment group (66.0 ± 3.1 pA, *n* = 106; Fig. 4*A*, two-tailed unpaired *t* test, *p* = 0.009). The excitability of neurons from mice in the ethanol treatment group was also lower at this range of positive current input, as shown by the input/output curve in Figure 4*B*. Here, the relationship between the amount of current injected over 500 ms and the resulting action potential frequency was shifted to the right for neurons from mice in the ethanol group compared with neurons from mice in the sucrose treatment group (two-way repeated-measures ANOVA for all data, interaction between effects of current and developmental treatment, *F*_(10,1930)_ = 4.7; *p* < 0.0001). Firing frequency was lower in neurons from mice in the ethanol treatment group on the rising phase of the input/output curve between 50 and 200 pA (two-way repeated-measures ANOVA, effect of current, *F*_(3,579)_ = 922.1; *p* < 0.0001; effect of developmental treatment, *F*_(1,193)_ = 4.9; *p* = 0.03; interaction between effects, *F*_(3,579)_ = 2.3; *p* = 0.07), and firing frequency was greater in neurons from the ethanol treatment group on the descending phase of the input/output curve between 350 and 500 pA (effect of current, *F*_(3,579)_ = 144.3; *p* < 0.0001; effect of developmental treatment, *F*_(1,193)_ = 4.2, *p* = 0.04; interaction between effects, *F*_(3,579)_ = 0.3; *p* = 0.8). Given the influence of developmental ethanol exposure on neuron excitability, we next analyzed effects of treatment on neuron afterhyperpolarization (AHP) by measuring the peak AHP after the end of the action potential trains generated in the input/output experiment. This measurement was performed at the 100-pA injection, where we observed an effect of developmental treatment on firing frequency (e.g., as shown in Fig. 4*C*) and at the 250-pA injection, where firing frequency was similar between treatment groups. As shown in Figure 4*D*, the peak AHP amplitude was greater in neurons from mice in the ethanol treatment group than in neurons from mice in the sucrose treatment group at both levels of current injection (two-way repeated-measures ANOVA on log-transformed data; effect of current, *F*_(1,174)_ = 56.4; *p* < 0.0001; effect of developmental treatment, *F*_(1,174)_ = 5.2; *p* = 0.02; interaction between effects, *F*_(1,174)_ = 0.04; *p* = 0.8; Mann-Whitney *U* test on raw data at each level of current injection, *p* < 0.04). AHP amplitudes after 100-pA current injection were 1.1 ± 0.1 mV (*n* = 92) for neurons in the sucrose treatment group and 1.4 ± 0.1 mV (*n* = 84) for neurons in the ethanol treatment group, and AHP amplitudes after 250-pA current injection were 1.4 ± 0.1 mV (*n* = 92) for neurons in the sucrose treatment group and 1.7 ± 0.1 mV (*n* = 84) for neurons in the ethanol treatment group.

### Developmental ethanol exposure increases the response to nicotinic receptor stimulation in prefrontal layer VI pyramidal neurons

**Figure 5.**
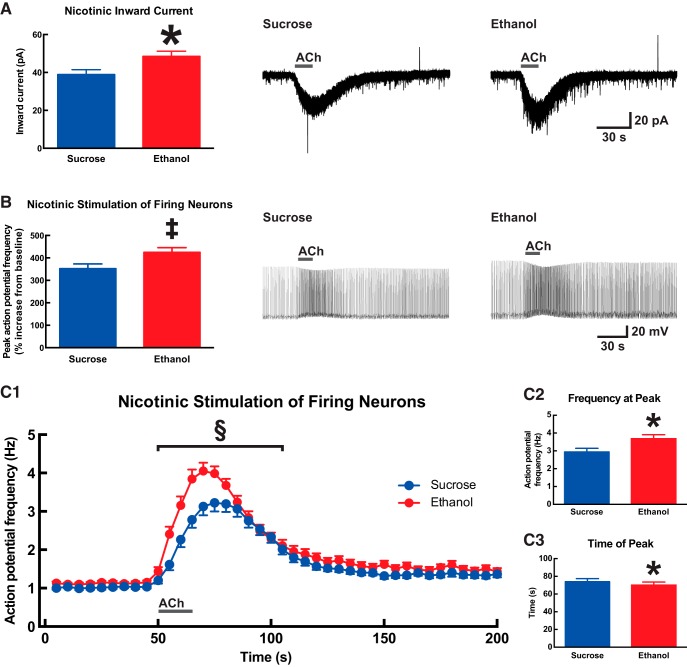
Developmental ethanol exposure increases nicotinic receptor function in adult medial prefrontal layer VI pyramidal neurons. ***A***, The peak inward current response to 1 mm acetylcholine (15 s in the presence of 200 nm atropine) was significantly greater in neurons from mice that were administered ethanol during development than in neurons from mice that were administered sucrose during development (two-tailed unpaired *t* test, *p = 0.01). Exemplary voltage-clamp traces are shown on the right for one neuron from each developmental treatment group. ***B***, For neurons that had been induced to fire action potentials by current injection, further nicotinic stimulation with 1 mm acetylcholine (15 s in the presence of 200 nm atropine) increased firing frequency to a greater degree in neurons from mice that were administered ethanol during development (Mann–Whitney *U* test, ^‡^*p* = 0.008). Exemplary current-clamp traces are shown on the right for one neuron from each developmental treatment group. The instantaneous firing frequency for this experiment is plotted against time in **C1**, where a significant effect of developmental treatment was observed during the acetylcholine response period (two-way ANOVA, ^§^*p* < 0.0001). Firing frequency peaked at a greater magnitude (**C2**, Mann–Whitney *U* test, *p = 0.01) and occurred at an earlier time (**C3**, two-tailed unpaired *t* test, *p = 0.01) in neurons from mice that were administered ethanol during development. Acetylcholine applications are indicated on all traces by a gray bar. All data are shown as mean ± 1 SEM.

Given the importance of cholinergic signaling within the mPFC (Passetti et al., 2000; Dalley et al., 2004; Parikh et al., 2007), and specifically at α4β2* nAChRs on mPFC layer VI pyramidal neurons (Guillem et al., 2011), for normal performance in attention tasks, we next sought to measure effects of developmental ethanol exposure on nAChR function. Whole-cell current responses were measured after the application of 1 mm ACh for 15 s in the presence of 200 nm atropine (to block muscarinic receptors). Such nicotinic responses are mediated in these neurons by α4β2* nAChRs (Kassam et al., 2008; Bailey et al., 2010; Poorthuis et al., 2013; Bloem et al., 2014). As shown in Figure 5*A*, nAChR current responses were significantly greater in neurons from mice in the ethanol treatment group (48.5 ± 2.7 pA, *n* = 62) than in neurons from mice in the sucrose treatment group (38.9 ± 2.6 pA, *n* = 58; two-tailed unpaired *t* test, *p* = 0.01). Nicotinic responses were next assessed in active neurons by injecting positive current to induce action potential firing at approximately 1 Hz, and then measuring the increase in firing rate in response to the application of 1 mm ACh for 15 s in the presence of 200 nm atropine. Here, the percent by which firing increased over baseline for each neuron was also greater in neurons from mice in the ethanol treatment group (425 ± 21%, *n* = 63) than in neurons from mice in the sucrose treatment group (352 ± 21%, *n* = 58; Fig. 5*B*, Mann–Whitney *U* test, *p* = 0.008). The magnitude and kinetics for instantaneous firing frequency in this experiment were also affected by developmental treatment (Fig. 5*C*). Firing frequency was greater during the ACh response period for neurons from mice in the ethanol treatment group (Fig. 5*C1* ; two-way ANOVA; effect of time, *F*_(11,1419)_ = 30.5; *p* < 0.0001; effect of developmental treatment, *F*_(1,1419)_ = 35.8; *p* < 0.0001). The peak firing frequency for each neuron was greater in neurons from mice in the ethanol treatment group (3.7 ± 0.2 Hz, *n* = 63) than in neurons from mice in the sucrose treatment group (2.9 ± 0.2 Hz, *n* = 58; Fig. 5*C2* ; two-tailed unpaired *t* test, *p* = 0.01). In addition, this peak ACh response occurred at an earlier time in neurons from mice in the ethanol treatment group (77.0 ± 2.4 s, *n* = 63) than in neurons from mice in the sucrose treatment group (81.5 ± 2.3 s, *n* = 58; Fig. 5*C3* ; Mann–Whitney *U* test, *p* = 0.01).

We also measured muscarinic ACh receptor (mAChR) function in the same mice and found no effect of developmental ethanol exposure. Whole-cell inward current responses after the application of 1 mm ACh for 15 s (in the presence of 3 μm DHβE to block α4β2* nAChRs; these neurons are not activated by α7 nAChRs) were 6.0 ± 0.7 pA (*n* = 28) for neurons from mice in the sucrose treatment group and 6.8 ± 0.4 pA (*n* = 35) for neurons from mice in the ethanol treatment group (two-tailed unpaired *t* test, *p* = 0.3). Muscarinic responses in active neurons were assessed by injecting positive current to induce action potential firing at approximately 1 Hz and then measuring the increase in firing rate in response to the application of 1 mm ACh for 15 s in the presence of 3 μm DHβE. The percentage increase in firing rate was not different between neurons from mice in the sucrose treatment group (328 ± 20%, *n* = 28) and neurons from mice in the ethanol treatment group (359 ± 22%, *n* = 35; Mann–Whitney *U* test, *p* = 0.5).

### Developmental ethanol exposure increases the response to AMPA receptor stimulation in prefrontal layer VI pyramidal neurons

**Figure 6.**
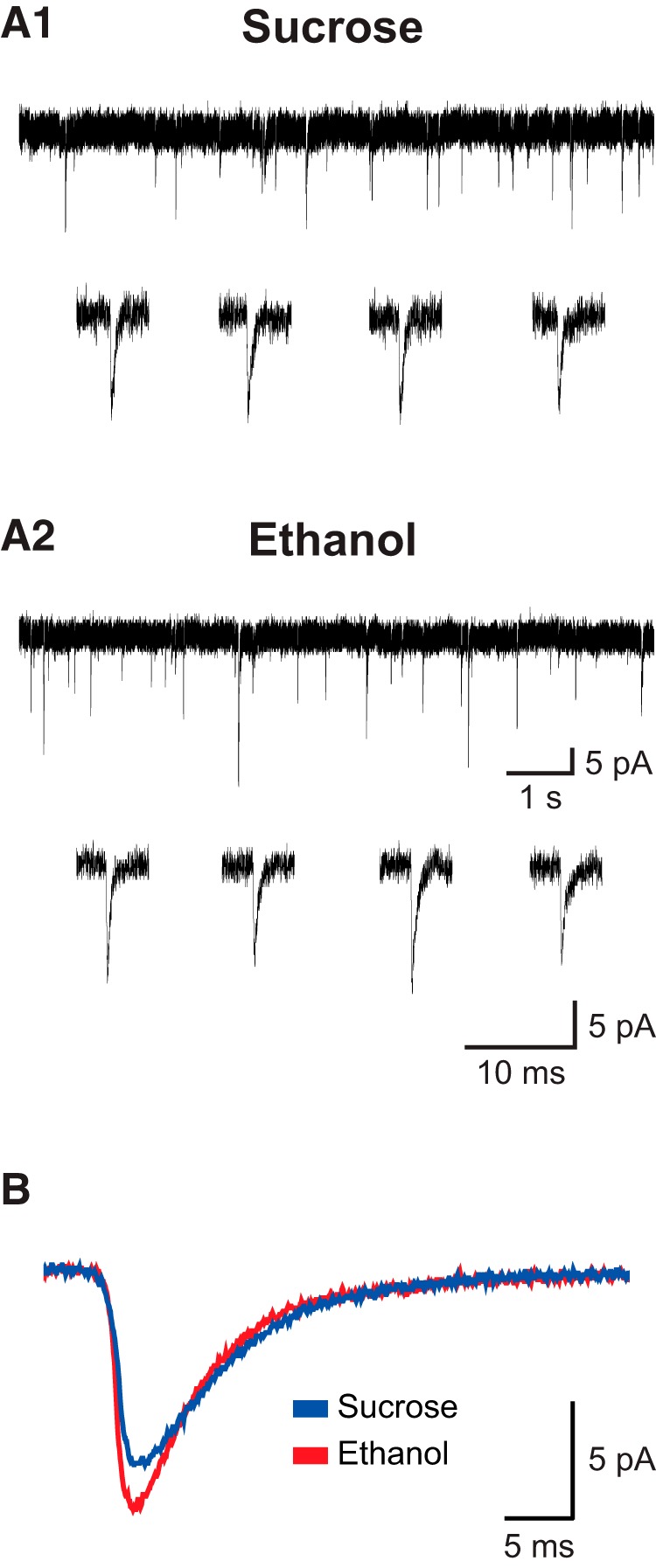
Exemplary traces of recorded glutamatergic sEPSCs. ***A***, Exemplary traces are shown for one neuron from the sucrose (**A1**) and ethanol (**A2**) developmental treatment groups held at –75 mV in voltage-clamp mode. For each neuron, traces of approximately 10 s in length are shown at the top, and four individual exemplary sEPSCs are shown at the bottom. ***B***, The average of 200 representative EPSC traces is shown for neurons from the sucrose (blue) and ethanol (red) developmental treatment groups. Data for the frequency, amplitude, and kinetics of sEPSCs in this study are shown in Table 4.

In performing the ACh experiments described above, we observed differences between experimental groups for the magnitude and kinetics of sEPSCs in mPFC layer VI pyramidal neurons. Spontaneous EPSCs were measured in voltage clamp mode for neurons held at –75 mV, which is near the measured equilibrium potential for chloride in our preparation of –73.5 mV. This nonpharmacological approach thus mitigates any influence of GABA_A_ receptor signaling and also is below the voltage threshold for NMDA glutamatergic receptor activation. Moreover, all sEPSCs in this preparation are blocked by the AMPA/kainate glutamatergic receptor competitive antagonist CNQX (data not shown). Data and statistical analyses for all neurons in this study are shown in Table 4. There was no effect of developmental treatment on the frequency of sEPSCs (Mann–Whitney *U* test, *p* = 0.6), although there was a trend toward a greater amplitude of sEPSCs in neurons from mice in the ethanol treatment group (*p* = 0.08). The onset kinetics for sEPSCs were significantly affected by developmental treatment, as the sEPSC rise time was shorter (*p* = 0.0008) and rise slope was greater (*p* = 0.02) in neurons from mice in the ethanol treatment group than in neurons from mice in the sucrose treatment group. The sEPSC decay time was not affected by developmental treatment (*p* = 0.9). Exemplar and average traces of recorded sEPSCs from neurons of each developmental treatment group are shown in Figure 6.

**Table 4.** Properties of sEPSCs in prefrontal layer VI pyramidal neurons.

Characteristic	Sucrose	Ethanol	*p*-value
Number of mice	14	16	
Number of neurons	98	104	
Frequency (Hz)	0.65 ± 0.06	0.68 ± 0.05	0.6
Amplitude (pA)	11.4 ± 0.3	12.4 ± 0.4	0.08
10–90 Rise (ms)	2.7 ± 0.1	2.3 ± 0.1	0.0008*
10–90 Slope (pA/ms)	–5.5 ± 0.3	–7.2 ± 0.5	0.02*
Decay (ms)	4.9 ± 0.2	5.0 ± 0.2	0.9

Data are presented as mean ± 1 SEM for neurons within each data set. Data sets were analyzed by Mann–Whitney *U* test. *Statistically significant (*p* < 0.05).

**Figure 7.**
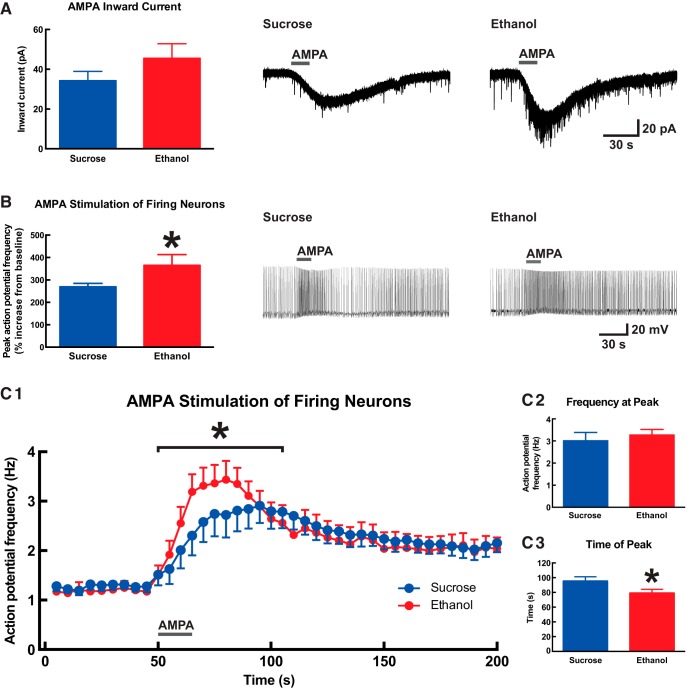
Developmental ethanol exposure increases AMPA receptor function in adult medial prefrontal layer VI pyramidal neurons. ***A***, The peak inward current response to 2 μm (S)-AMPA (15 s) was not significantly different between neurons from mice that were administered ethanol during development and neurons from mice that were administered sucrose during development (Mann–Whitney *U* test, *p* = 0.1). Exemplary voltage-clamp traces are shown on the right for one neuron from each developmental treatment group. ***B***, For neurons that had been induced to fire action potentials by current injection, further glutamatergic stimulation with 2 μm (S)-AMPA (15 s) increased firing frequency to a greater degree in neurons from mice that were administered ethanol during development (two-tailed unpaired *t* test, *p = 0.04). Exemplary current-clamp traces are shown on the right for one neuron from each developmental treatment group. The instantaneous firing frequency for this experiment is plotted against time in **C1**, where a significant effect of developmental treatment was observed during the (S)-AMPA response period (two-way repeated-measures ANOVA, *p = 0.02). The peak firing frequency was not significantly different between developmental treatment groups (**C2**, two-tailed unpaired *t* test, *p* = 0.6) although it did occur at an earlier time in neurons from mice that were administered ethanol during development (**C3**, two-tailed unpaired *t* test, *p = 0.047). AMPA applications are indicated on all traces by a gray bar. All data are shown as mean ± 1 SEM.

Given the observed effects of developmental binge-pattern ethanol exposure on AMPA/kainate-mediated EPSCs and the importance of glutamatergic signaling within the mPFC for attention (Murphy et al., 2005; Quarta et al., 2007; Parikh et al., 2008, 2010; Howe et al., 2010), we next measured AMPA receptor function directly in mPFC layer VI pyramidal neurons. Experiments were performed on a subset of mice from study (ethanol, *n* = 6; sucrose, *n* = 9). We first measured whole-cell current responses after 15-s application of 2 μm (S)-AMPA and found the difference between treatment group means to be similar in magnitude to that for nicotinic currents [Fig. 7*A*; ethanol: 45.5 ± 7.4 pA (*n* = 12); sucrose: 34.2 ± 4.7 pA (*n* = 17)], although this difference was not significant (Mann–Whitney *U* test, *p* = 0.1). Excitatory responses to AMPA were next assessed in active neurons by injecting positive current to induce action potential firing at approximately 1 Hz, and then measuring the increase in firing rate after 15-s application of 2 μm (S)-AMPA. The percentage by which firing increased over baseline for each neuron was significantly greater in neurons from mice in the ethanol treatment group (365 ± 48%, *n* = 12) than in neurons from mice in the sucrose treatment group (270 ± 15%, *n* = 16; Fig. 7*B*; two-tailed unpaired *t* test, *p* = 0.04). The timing for AMPA responses in this experiment was also affected by developmental treatment (Fig. 7*C*). Firing frequency was greater during the AMPA response period for neurons from mice in the ethanol treatment group (Fig. 7*C1* ; two-way ANOVA; effect of time, *F*_(11,311)_ = 4.0; *p* < 0.0001; effect of developmental treatment, *F*_(1,311)_ = 5.4; *p* = 0.02). The peak firing frequency for each neuron was not significant between neurons from mice in the ethanol treatment group (3.3 ± 0.2 Hz, *n* = 12) and neurons from mice in the sucrose treatment group (3.0 ± 0.4 Hz, *n* = 16; Fig. 7*C2* ; two-tailed unpaired *t* test, *p* = 0.6). However, the peak response to AMPA occurred at an earlier time in neurons from mice in the ethanol treatment group (79.8 ± 4.9 s, *n* = 12) than in neurons from mice in the sucrose treatment group (96.1 ± 5.7 s, *n* = 16; Fig. 7*C3* ; *p* = 0.047).

### Developmental ethanol exposure disrupts the relationship between prefrontal nicotinic receptor function and performance on an attention task

The analysis of neuron function and performance on the 5-CSRTT within the same experimental animals provided the opportunity to determine whether specific properties of mPFC layer VI pyramidal neurons correlate with attention performance. Neuron electrophysiological properties were compared with two measures of attention processing that were negatively affected by developmental ethanol exposure: (i) accuracy percentage at the 8-s stimulus duration (full correlation data are presented in Table 5) and (ii) percentage of omissions at the 1-s stimulus duration (full correlation data are presented in Table 6). Mice in the sucrose group showed a positive correlation between nicotinic inward currents (data from Fig. 5*A*) and accuracy percentage (data from Fig. 2*C*; *p* = 0.02), and also a strong trend toward a positive correlation between nicotinic stimulation of firing neurons (data from Fig. 5*B*) and accuracy percentage (data from Fig. 2*C*; *p* = 0.06). This indicates that mice in the sucrose group with greater layer VI neuron α4β2* nAChR function performed with greater accuracy on the 5-CSRTT task. In contrast, mice in the ethanol group showed no correlation between nicotinic inward currents and accuracy (*p* = 0.4), or between nicotinic simulation of firing neurons and accuracy (*p* = 0.8). Mice in the sucrose group showed a negative correlation between nicotinic inward currents (data from Fig. 5*A*) and omissions (data from Fig. 2*D*; *p* = 0.05), and also a negative correlation between nicotinic stimulation of firing neurons (data from Fig. 5*B*) and omissions (data from Fig. 2*D*; *p* = 0.03). This indicates that mice in the sucrose group with greater layer VI neuron α4β2* nAChR function performed with fewer omissions on the 5-CSRTT task. In contrast, mice in the ethanol group showed no correlation between nicotinic inward currents and omissions (*p* = 0.9), or between nicotinic stimulation of firing neurons and omissions (*p* = 0.9). There were no additional correlations in this study between any other electrophysiological measure and attention performance, suggesting that observed relationships between mPFC layer VI neuron function and attention performance in the sucrose group were selective to those involving nicotinic signaling.

**Table 5.** Correlation analysis comparing electrophysiological properties of prefrontal layer VI pyramidal neurons and accuracy percentage at the 8-s stimulus duration in the 5-CSRTT.

Correlation versus accuracy	Sucrose		Ethanol	
	Pearson *r*	*p*-value	Pearson *r*	*p*-value
Resting membrane potential	–0.37	0.19	0.08	0.78
Capacitance	0.28	0.34	–0.09	0.73
Input resistance	–0.41	0.14	–0.01	0.99
Spike amplitude	0.25	0.38	0.29	0.27
Rheobase	0.51	0.06	0.22	0.52
Receptor-mediated inward currents				
Nicotinic	0.61	0.02	0.21	0.43
Muscarinic	0.03	0.92	0.46	0.07
AMPA glutamatergic	–0.06	0.87	–0.16	0.77
Receptor-mediated stimulation of firing neurons				
Nicotinic	0.51	0.06	0.08	0.78
Muscarinic	0.41	0.17	0.27	0.31
AMPA glutamatergic	0.15	0.72	–0.25	0.64

**Table 6.** Correlation analysis comparing electrophysiological properties of prefrontal layer VI pyramidal neurons and percentage of omissions at the 1-s stimulus duration in the 5-CSRTT.

Correlation versus omissions	Sucrose		Ethanol	
	Pearson *r*	*p*-value	Pearson *r*	*p*-value
Resting membrane potential	0.46	0.09	0.25	0.35
Capacitance	0.31	0.27	0.23	0.40
Input resistance	0.02	0.95	0.25	0.35
Spike amplitude	0.33	0.24	0.33	0.21
Rheobase	–0.17	0.55	–0.01	0.98
Receptor-mediated inward currents				
Nicotinic	–0.52	0.05	0.03	0.93
Muscarinic	0.09	0.77	–0.08	0.78
AMPA glutamatergic	0.51	0.16	0.31	0.55
Receptor-mediated stimulation of firing neurons				
Nicotinic	–0.58	0.03	0.02	0.93
Muscarinic	–0.28	0.36	0.14	0.62
AMPA glutamatergic	0.10	0.81	0.27	0.61

## Discussion

This study provides novel insight into the long-term consequences of developmental binge-pattern ethanol exposure on prefrontal attention systems. We found that adult mice exposed to ethanol during development showed decreased performance on the 5-CSRTT for visual attention, as they performed with lower accuracy when first learning the task and with a higher rate of omissions under conditions that required the greatest attentional demand. We then measured the function of pyramidal neurons located within mPFC layer VI of these same experimental animals because cholinergic excitation of this neuronal population is necessary for normal attention performance (Dalley et al., 2004; Parikh et al., 2007; Guillem et al., 2011), these neurons are strongly excited by nAChRs to support attention (Kassam et al., 2008; Bailey et al., 2010; Guillem et al., 2011), and developmental ethanol exposure likely alters the function of nAChRs within cognitive systems (Nagahara and Handa, 1999). Here, we found that developmental ethanol exposure dysregulated layer VI pyramidal neurons by decreasing intrinsic excitability and increasing responses to stimulation of both α4β2* nAChRs and AMPA glutamate receptors. These effects were observed approximately 8 months after ethanol exposure, demonstrating the persistence of ethanol’s influence on developing prefrontal circuitry. The developmental ethanol exposure paradigm in this study modeled a binge pattern of administration characterized by daily oral ethanol doses that achieved relatively high BEC values, as opposed to alternative approaches that provide sustained access to ethanol in drinking water or liquid diet that typically result in lower BEC values. Self-reported survey data from North America suggests that 25–42% of women drink alcohol during the first trimester, including 8–20% who binge drink, and that 8% of women drink alcohol in the third trimester, including 1% who binge drink (Ethen et al., 2009; Alshaarawy et al., 2016). In humans (May et al., 2013; Flak et al., 2014) and rodents (West et al., 1989), the degree of teratogenic damage to the brain is greater after binge ethanol consumption (higher BEC) than after mild to moderate ethanol consumption (lower BEC), so it will be important in future work to compare the mPFC data from this study with that following a nonbinge pattern of developmental ethanol exposure.

### Developmental ethanol exposure and attention

Mice from the ethanol treatment group required more days to meet training criteria on the 5-CSRTT at the initial 8-s stimulus duration because they performed with lower accuracy. Indeed, although mice from the sucrose treatment group performed with an average of 82.1 ± 1.8% accuracy across all training days at 8 s, the average value of 75.6 ± 2.0% for mice in the ethanol treatment group falls below required criterion cutoff of 80%. Although reduced accuracy on the 5-CSRTT is considered to indicate impaired attention (Robbins, 2002), it is also possible that impaired learning contributed to this result, because this was the first opportunity for mice to perform the full version of the task. In support of this learning hypothesis, note that mice from both treatment groups required more time to complete 60 trials, committed more premature responses, and performed with a longer correct response latency at the 8-s stimulus duration than at subsequent stimulus durations.

Mice from both groups appear to have learned the task equally well after advancing from the 8-s stimulus duration, as they met advancement criteria near the minimum number of days from the 4- to 1.4-s stimulus durations. An effect of developmental treatment then re-emerged at the lowest stimulus duration tested of 1 s, as mice from the ethanol treatment group again required more days to meet training criteria. This dramatic increase in days to criteria for mice from the ethanol treatment group likely resulted from the average percent omissions across all training days of 17.5 ± 1.2 (95% confidence interval = 14.9 to 20.2%), which fell close to the criterion cutoff of 20%. Errors of omission on the 5-CSRTT increased for both treatment groups as stimulus duration decreased. However, percentage of omissions was greater overall for mice from the ethanol treatment group and significantly greater by *post hoc* analysis at the lowest 1.2- and 1-s stimulus durations. In the absence of treatment effects on correct response latency or reward collection latency, which incorporate potentially confounding sensory and motor functions in addition to overall motivation (Robbins, 2002), this suggests that mice from the ethanol treatment group exhibited impaired global attention processing (Mar et al., 2013) that was most pronounced at the lowest stimulus durations requiring the greatest attentional demand. This effect of developmental ethanol exposure to increase omissions is striking, because the 5-CSRTT version used in this study required mice to initiate each trial after a fixed ITI to self-regulate session pace (Mar et al., 2013) instead of the more widely used 5-CSRTT version originally developed for rats where trials following correct responses/reward collection are automatically initiated (Bari et al., 2008). We used this strategy because mice generally perform this task with a greater percentage of omissions than rats (Fletcher et al., 2007; Bailey et al., 2010; Mar et al., 2013), and its use suggests that the higher rate of omissions in the ethanol treatment group cannot be attributed to mice taking longer to collect food reward or missing initiation of the next trial. Visual sensory processing was not directly tested in this study, so it remains possible that alterations to visual acuity influenced percent omissions in ethanol-treated mice. Evidence against this interpretation includes the lack of treatment effects on percent accuracy below the 8 s stimulus duration or on correct response latency at any stimulus duration, and a published finding that developmental ethanol exposure does not affect learning of a visual discrimination task (Marquardt et al., 2014).

Effects of developmental ethanol exposure on 5-CSRTT performance in this current study are consistent with the attention deficit profile observed in children affected by FASD. Studies in children exposed to ethanol prenatally find impairments on continuous performance tasks for sustained attention, characterized consistently by an increased omission rate (Brown et al., 1991; Lee et al., 2004; Infante et al., 2015). Moreover, although impulsivity is a major component of the ADHD behavioral profile, it is not as prevalent within the FASD behavioral profile (Brown et al., 1991; Infante et al., 2015), and we also found no treatment effect on premature responding in this study. Developmental ethanol exposure via liquid diet was recently reported to impart similar effects on rat attention performance. One study found developmental ethanol to increase percentage of omissions on the 5-CSRTT (Brys et al., 2014), whereas another study found this effect only in conjunction with developmental stress (Comeau et al., 2014), and neither study observed changes to premature responding. These studies, together with our results, recapitulate the main components of the attention deficit profile in FASD, confirming that rodents are appropriate models to determine underlying neuronal mechanisms.

### Developmental ethanol exposure and prefrontal layer VI pyramidal neurons

We studied mPFC layer VI pyramidal neurons because approximately 40% of this neuronal population contributes to attention circuitry through the modulation of corticothalamic signal gain (Gabbott et al., 2005; Zikopoulos and Barbas, 2006; Olsen et al., 2012; Sherman, 2016) and because of the potential for developmental ethanol exposure to dysregulate nicotinic support of these processes, as described above. The recording of retrograde-labeled corticothalamic projection neurons would have provided a more restricted analysis of this mPFC layer VI pyramidal neuron subtype only, allowing for direct comparisons between corticothalamic signaling and attention performance. The random sampling of pyramidal neurons that we used within layer VI alternatively allowed for the incorporation of additional pyramidal neuron subtypes that may also contribute to cognitive functions, including attention, through their projections within the medial prefrontal cortex itself and also to subcortical brain regions including the striatum, hypothalamus, and amygdala (Gabbott et al., 2005; Hoover and Vertes, 2007). It is important to consider the potential for developmental ethanol exposure to alter the laminar organization of the mature mPFC, which could have led to the sampling of distinct populations of pyramidal neurons within layer VI of mice from each treatment group. Although reports in mouse (Smiley et al., 2015) and guinea pig (Bailey et al., 2004) demonstrate normal cortical layering for primary motor/sensory cortices after developmental ethanol exposure, a detailed histological analysis of the mPFC is required to confirm these findings for this associative cortical region.

Both passive and active basic electrophysiological properties of layer VI neurons were altered by developmental binge-pattern ethanol exposure, resulting in decreased neuronal function. Decreased capacitance and a trend toward increased input resistance suggest smaller neurons in mice from the ethanol group (Dégenètais et al., 2002), which alone could increase their passive response to positive input. However, decreased active function of these same neurons was evidenced by increased rheobase and decreased firing frequency in the range of 50- to 200-pA positive current injection. This decreased firing frequency may result from the larger AHP amplitudes measured in neurons from mice in the ethanol group, which in turn are influenced by BK and SK calcium-activated potassium channels (Faber and Sah, 2003; Pedarzani and Stocker, 2008). Although acute ethanol exposure decreases neuronal excitability through BK channels (Martin et al., 2004; Dopico et al., 2014) and increases neuronal excitability through SK channels (Brodie et al., 1999; Korkotian et al., 2013), long-term consequences of developmental ethanol exposure on these channels and their specific roles in mPFC layer VI neuron excitability remain to be determined.

The effect of developmental binge-pattern ethanol exposure to decrease intrinsic excitability of mPFC layer VI neurons is contrasted by an increase in function for excitatory nAChRs and AMPA receptors. Upregulated receptor function at the neuronal level could result from increased expression of subunit protein or from increased function of individual receptors. Pyramidal neurons in mPFC layer VI are excited directly by α4β2* nAChRs that may exhibit augmented function from posttranslational modification (Henderson and Lester, 2015). A proportion of α4β2* nAChRs in layer VI neurons contain the α5 accessory subunit that increases receptor-mediated currents when present (Wada et al., 1990; Kassam et al., 2008; Bailey et al., 2010; Poorthuis et al., 2013), so augmented function at the neuronal level may result from the selective increase in α5 subunit expression or incorporation into receptors. Although mPFC layer VI neurons in untreated animals are not believed to express functional α7 subunit–containing nAChRs (Kassam et al., 2008; Poorthuis et al., 2013), it is possible that the expression or function of this nAChR subtype is selectively upregulated after developmental ethanol exposure. Evidence against this possibility can be found in a follow-up study currently underway in our laboratory, in which the inhibition of α7 subunit–containing nAChRs using methyllycaconitine did not affect nAChR function in mPFC layer VI neurons after developmental ethanol exposure (data not shown). Given the large number of nAChR subunit genes and isoform combinations in the brain, it would be advantageous to complete a comprehensive analysis of subunit expression and isoform content within each neuron type of the mPFC after developmental binge-pattern ethanol exposure, to fully determine mechanisms underlying the augmented excitatory responses to ACh observed in this study. To our knowledge, the single study to examine effects of developmental ethanol exposure on nAChR content found decreased brainstem receptor number as a function of increased prenatal ethanol exposure in children who had died of sudden infant death syndrome (Duncan et al., 2008).

We observed faster activation kinetics of sEPSCs in mPFC layer VI neurons after developmental binge-pattern ethanol exposure, which may reflect the faster activation of AMPA- versus kainate-mediated sEPSCs (Cossart et al., 2002), suggesting an increased AMPA:kainate receptor ratio in these neurons. We also observed a trend toward increased sEPSC amplitude and significantly increased responses to direct AMPA receptor activation, which all suggest increased AMPA receptor subunit protein expression, altered subunit/splice variant composition (Lambolez et al., 1996), or altered association with transmembrane regulatory proteins (Kato et al., 2010). Previous studies in rat found that AMPA receptors were not affected in the hippocampus (Martin et al., 1992) and had decreased expression in whole cerebral cortex (Bellinger et al., 2002) after developmental ethanol exposure, which may indicate a species difference or the specificity of our observed results to mPFC layer VI pyramidal neurons. A more detailed examination of glutamatergic neurotransmission at these neurons could address these remaining questions. Potential analyses include the measurement of EPSCs that are activity-dependent (evoked EPSCs) and activity-independent (mini-EPSCs), the measurement of AMPA/Kainate/NMDA receptor function, and the analysis of AMPA/Kainate/NMDA receptor expression and biochemistry.

It should be noted that for all electrophysiological data that are determined to be significantly affected by developmental treatment, significance is also attained when the mouse is used as the unit of determination. One exception is the AMPA receptor data presented in Figure 7, because these experiments were performed only in approximately one-half the mice that were used in this study, and their analyses did not attain sufficient statistical power.

### Implications for prefrontal cholinergic signaling in attention

Cholinergic signaling within the mPFC (Passetti et al., 2000; Dalley et al., 2004; Parikh et al., 2007), and specifically at α4β2* nAChRs on layer VI pyramidal neurons (Guillem et al., 2011), is critical for normal attention. We provide further evidence here in sucrose/control mice for the role of α4β2* nAChRs on layer VI neurons to support attention processing, through a selective combination of correlations between receptor function and performance on the 5-CSRTT. Upregulated receptor function, impaired performance on the 5-CSRTT, and a lack of correlations between the two after developmental binge-pattern ethanol exposure suggest that this treatment may disrupt the ability of nicotinic signaling at mPFC layer VI neurons to support attention processing. Confirmation of a causal link between dysregulated nicotinic signaling at mPFC layer VI neurons and decreased attention performance after developmental binge-pattern ethanol exposure would need to be performed in future studies. For example, selective manipulation of nAChR function on mPFC layer VI neurons could be performed in control and developmental ethanol-treated rodents while they are performing the 5-CSRTT. It is important to note that the original rodent lesioning studies demonstrating a role for the mPFC to support performance on the 5-CSRTT were not performed using touchscreen equipment (Muir et al., 1996; Chudasama et al., 2003; Dalley et al., 2004). Although these lesioning studies have not yet been repeated using the touchscreen version of the 5-CSRTT, our results add to a growing body of literature providing correlational evidence that the mPFC also supports mouse performance on this version of the task (McTighe et al., 2013; Nilsson et al., 2016).

We did not expect decreased performance on the 5-CSRTT in the ethanol treatment group to be associated with increased nAChR function in mPFC layer VI neurons because signaling at this receptor normally supports attention processing. However, it should be noted that these neurons display a generally dysregulated profile that also includes decreased intrinsic excitability, and it is not currently known whether nAChR function is upregulated to compensate for decreased intrinsic excitability or intrinsic excitability is downregulated to compensate for increased nAChR function. Moreover, the net effect of these neurophysiological outcomes of developmental ethanol exposure on the function of mPFC layer VI neurons within *in vivo* attention circuitry is not known. It should also be noted that agonist augmentation of nAChR function in rodents exhibits a U-shaped curve for performance in attention tasks (McGaughy et al., 1999; Hahn et al., 2002; Hahn et al., 2003), suggesting another explanation for our data that nAChR function within mPFC layer VI neurons requires a tight operational range to optimally support attention. In conclusion, our findings demonstrate novel mechanisms underlying dysregulation of prefrontal attention circuitry after developmental binge-pattern ethanol exposure and suggest the remediation of mPFC layer VI neuron function as a potential therapeutic target to mitigate attention deficits in FASD.

## Synthesis

The decision was a result of the Reviewing Editor Tim Bussey and the peer reviewers coming together and discussing their recommendations until a consensus was reached. A fact-based synthesis statement explaining their decision and outlining what is needed to prepare a revision is listed below. The following reviewers agreed to reveal their identity: Carlos Valenzuela, Christopher Heath

reviewers The and I felt that your study was interesting, well-written, and valuable. There are a few issues, however, that still need to be addressed. A main issue that came out of the reviews and our consultation is the "unit of determination" (litter, pup, cell) used in the analysis of each experiment. In particular, it needs to be confirmed that averaged recordings are from slices from a pup and the pup is the unit of determination. Both reviewers also agree that it should be made clear what exactly the ethanol exposure paradigm is modelling (e.g., binge drinking, see reviewer 1's comment 3 below; FASD, see reviewer 2). Both reviewers also agree that the correlations shown in Fig 8 add little to the report and should be removed. Finally, each reviewer has more minor comments that need to be addressed. If these changes are thorough and clear, I hope to be able to evaluate them myself without sending the manuscript back to the reviewers. Both reviews are reproduced in full below.

Reviewer 1

The paper of Louth et al reports that chronic prenatal ethanol exposure impaired performance on the five-choice serial reaction time test. Slice electrophysiological experiments revealed functional alterations in pyramidal neurons in layer VI of the medial prefrontal cortex, including increased responses to agonists of nAChRs and AMPA receptors. The paper is well-written and results are compelling and interesting. This paper was previously submitted for consideration to J. Neurosci and has now been transferred to eNeuron. The authors appropriately addressed the concerns raised by the reviewers of the original submission. There are only a few issues for the authors' consideration:

1. The shunting effects of GABAA receptor activation (including via tonic currents) should still influence neuronal function even at the chloride equilibrium potential. The investigators may want to comment on whether this could affect interpretation of results.

2. In my opinion, the correlations shown in Fig 8 add little to the paper and removing them would not detract from the study. In order to provide a conclusive link between the alterations in receptor function and the behavioral deficits, selective manipulation of the function of this receptors in Layer VI pyramidal neurons would be required. The behavioral results could be presented as part of the general characterization of the ethanol exposure paradigm and in the discussion it could be acknowledged that receptor alterations may contribute to these effects but that additional experiments would be required to address this issue.

3. The ethanol exposure paradigm appears to model binge ethanol exposure. The authors may want to emphasize this a bit more, including discussion of % of women that binge drink during pregnancy and whether this pattern of ethanol exposure is more damaging to the CNS than non-binge exposure.

4. Please clarify and justify the choice of unit of determination (litter, pup, cell) for each experiment. In Tables 3 and 4, both the number of mice and neurons are provided and it needs to be clarified which one of these was used for statistical analyses.

5. Fig 6. Please also provide average traces illustrating differences in rise time and/or decay time.

Reviewer 2

Taken together, the data presented in this manuscript are timely and impactful, providing a well-executed behavioural and electrophysiological characterisation of a mouse model of perinatal ethanol exposure upon which a number of future studies can be based.

In my view the authors have also effectively addressed the comments made by the Reviewers of the previous version of the manuscript. The lack of identification of the targets for the recorded layer VI neurons does weaken the interpretation of the findings with respect to attentional mechanisms, given the acknowledged heterogeneity in these projections. However, this should be balanced with the existing data, the detailed discussion of the caveats given by the authors and the length and complexity of the procedure necessary to generate the additional animals to address this issue. As such, I believe the manuscript is suitable for publication in eNeuro, although there are a small number of issues the authors should be asked to address prior to final acceptance.

Major concerns:

1. The authors note that the mice involved in the study were drawn from multiple litters but that there wasn't a 1:1 correlation between the number of mice used and the number of litters produced. Can the authors specify the maximum number of animals drawn from any one litter to ensure that the reported effects are not confounded by an overrepresentation of a limited number of litters?

Minor concerns:

1. In the significance statement the authors state that the perinatal ethanol exposure model used in the manuscript is a 'mouse model of FASD'. Such a statement is not made elsewhere in the manuscript and no literature support is provided. Can the authors confirm that this exposure paradigm is considered an FASD model?

2. In the Introduction the authors note (on lines 102 - 104) that 40% of corticothalamic neurons project to the thalamic reticular nucleus - is this not the mediodorsal projection?

3. Can the authors explain why the BEC determination was performed using mice exposed to ethanol between P4 and P10 and not P4 and P14 to be consistent with the mice used in the rest of the study?

4. The frequency and duration of tone used in the 5-choice serial reaction time task (line 213) should be added.

## References

[B1] Aistrup GL, Marszalec W, Narahashi T (1999) Ethanol modulation of nicotinic acetylcholine receptor currents in cultured cortical neurons. Mol Pharmacol 55:39–49. 9882696 10.1124/mol.55.1.39

[B2] Alshaarawy O, Breslau N, Anthony JC (2016) Monthly estimates of alcohol drinking during pregnancy: United States, 2002-2011. J Stud Alcohol Drugs 77:272–276. 10.15288/jsad.2016.77.272 26997185 PMC4803659

[B3] Bailey CD, Alves NC, Nashmi R, De Biasi M, Lambe EK (2012) Nicotinic alpha5 subunits drive developmental changes in the activation and morphology of prefrontal cortex layer VI neurons. Biol Psychiatr 71:120–128. 10.1016/j.biopsych.2011.09.011 22030359 PMC3788582

[B4] Bailey CD, Brien JF, Reynolds JN (2001) Chronic prenatal ethanol exposure increases GABAA receptor subunit protein expression in the adult guinea pig cerebral cortex. J Neurosci 21:4381–4389. 11404424 10.1523/JNEUROSCI.21-12-04381.2001 PMC6762773

[B5] Bailey CD, Brien JF, Reynolds JN (2004) Chronic prenatal ethanol exposure alters the proportion of GABAergic neurons in layers II/III of the adult guinea pig somatosensory cortex. Neurotoxicol Teratol 26:59–63. 10.1016/j.ntt.2003.08.002 15001214

[B6] Bailey CD, De Biasi M, Fletcher PJ, Lambe EK (2010) The nicotinic acetylcholine receptor alpha5 subunit plays a key role in attention circuitry and accuracy. J Neurosci 30:9241–9252. 10.1523/JNEUROSCI.2258-10.2010 20610759 PMC3004929

[B7] Bari A, Dalley JW, Robbins TW (2008) The application of the 5-choice serial reaction time task for the assessment of visual attentional processes and impulse control in rats. Nat Protoc 3:759–767. 10.1038/nprot.2008.41 18451784

[B8] Bellinger FP, Davidson MS, Bedi KS, Wilce PA (2002) Neonatal ethanol exposure reduces AMPA but not NMDA receptor levels in the rat neocortex. Dev Brain Res 136:77–84. 10.1016/S0165-3806(02)00363-2 12036520

[B9] Bhatara V, Loudenberg R, Ellis R (2006) Association of attention deficit hyperactivity disorder and gestational alcohol exposure: an exploratory study. J Atten Disord 9:515–522. 10.1177/1087054705283880 16481668

[B10] Bloem B, Poorthuis RB, Mansvelder HD (2014) Cholinergic modulation of the medial prefrontal cortex: the role of nicotinic receptors in attention and regulation of neuronal activity. Front Neural Circuits 8:17. 10.3389/fncir.2014.00017 24653678 PMC3949318

[B11] Brocardo PS, Boehme F, Patten A, Cox A, Gil-Mohapel J, Christie BR (2012) Anxiety- and depression-like behaviors are accompanied by an increase in oxidative stress in a rat model of fetal alcohol spectrum disorders: protective effects of voluntary physical exercise. Neuropharmacology 62:1607–1618. 10.1016/j.neuropharm.2011.10.006 22019722

[B12] Brodie MS, McElvain MA, Bunney EB, Appel SB (1999) Pharmacological reduction of small conductance calcium-activated potassium current (SK) potentiates the excitatory effect of ethanol on ventral tegmental area dopamine neurons. J Pharmacol Exp Ther 290:325–333. 10381795

[B13] Brown RT, Coles CD, Smith IE, Platzman KA, Silverstein J, Erickson S, Falek A (1991) Effects of prenatal alcohol exposure at school age. II. Attention and behavior. Neurotoxicol Teratol 13:369–376. 1921916 10.1016/0892-0362(91)90085-b

[B14] Brys I, Pupe S, Bizarro L (2014) Attention, locomotor activity and developmental milestones in rats prenatally exposed to ethanol. Int J Dev Neurosci 38:161–168. 10.1016/j.ijdevneu.2014.08.007 25192749

[B15] Cardoso RA, Brozowski SJ, Chavez-Noriega LE, Harpold M, Valenzuela CF, Harris RA (1999) Effects of ethanol on recombinant human neuronal nicotinic acetylcholine receptors expressed in Xenopus oocytes. J Pharmacol Exp Ther 289:774–780. 10215652

[B16] Chudasama Y, Passetti F, Rhodes SE, Lopian D, Desai A, Robbins TW (2003) Dissociable aspects of performance on the 5-choice serial reaction time task following lesions of the dorsal anterior cingulate, infralimbic and orbitofrontal cortex in the rat: differential effects on selectivity, impulsivity and compulsivity. Behav Brain Res 146:105–119. 14643464 10.1016/j.bbr.2003.09.020

[B17] Chudley AE, Conry J, Cook JL, Loock C, Rosales T, LeBlanc N (2005) Fetal alcohol spectrum disorder: Canadian guidelines for diagnosis. CMAJ 172:S1–S21. 10.1503/cmaj.1040302 15738468 PMC557121

[B18] Comeau WL, Winstanley CA, Weinberg J (2014) Prenatal alcohol exposure and adolescent stress: unmasking persistent attentional deficits in rats. Eur J Neurosci 40:3078–3095. 10.1111/ejn.12671 25059261 PMC4189965

[B19] Cossart R, Epsztein J, Tyzio R, Becq H, Hirsch J, Ben-Ari Y, Crépel V (2002) Quantal release of glutamate generates pure kainate and mixed AMPA/kainate EPSCs in hippocampal neurons. Neuron 35:147–159. 12123615 10.1016/s0896-6273(02)00753-5

[B20] Cui ZJ, Zhao KB, Zhao HJ, Yu DM, Niu YL, Zhang JS, Deng JB (2010) Prenatal alcohol exposure induces long-term changes in dendritic spines and synapses in the mouse visual cortex. Alcohol Alcohol 45:312–319. 10.1093/alcalc/agq036 20543181

[B21] Dalley JW, Theobald DE, Bouger P, Chudasama Y, Cardinal RN, Robbins TW (2004) Cortical cholinergic function and deficits in visual attentional performance in rats following 192 IgG-saporin-induced lesions of the medial prefrontal cortex. Cereb Cortex 14:922–932. 10.1093/cercor/bhh052 15084496

[B22] Dégenètais E, Thierry AM, Glowinski J, Gioanni Y (2002) Electrophysiological properties of pyramidal neurons in the rat prefrontal cortex: an in vivo intracellular recording study. Cereb Cortex 12:1–16. 10.1093/cercor/12.1.1 11734528

[B23] Doig J, McLennan JD, Gibbard WB (2008) Medication effects on symptoms of attention-deficit/hyperactivity disorder in children with fetal alcohol spectrum disorder. J Child Adolesc Psychopharmacol 18:365–371. 10.1089/cap.2007.0121 18759646

[B24] Dopico AM, Bukiya AN, Martin GE (2014) Ethanol modulation of mammalian BK channels in excitable tissues: molecular targets and their possible contribution to alcohol-induced altered behavior. Front Physiol 5:466. 10.3389/fphys.2014.00466 25538625 PMC4256990

[B25] Duncan JR, Randall LL, Belliveau RA, Trachtenberg FL, Randall B, Habbe D, Mandell F, Welty TK, Iyasu S, Kinney HC (2008) The effect of maternal smoking and drinking during pregnancy upon 3H‐nicotine receptor brainstem binding in infants dying of the sudden infant death syndrome: initial observations in a high risk population. Brain Pathol 18:21–31. 10.1111/j.1750-3639.2007.00093.x 17924983 PMC8095492

[B26] Ethen MK, Ramadhani TA, Scheuerle AE, Canfield MA, Wyszynski DF, Druschel CM, Romitti PA, National Birth Defects Prevention S (2009) Alcohol consumption by women before and during pregnancy. Matern Child Health 13:274–285. 10.1007/s10995-008-0328-2 18317893 PMC6090563

[B27] Faber ES, Sah P (2003) Calcium-activated potassium channels: multiple contributions to neuronal function. Neurosci 9:181–194. 10.1177/1073858403009003011 15065814

[B28] Flak AL, Su S, Bertrand J, Denny CH, Kesmodel US, Cogswell ME (2014) The association of mild, moderate, and binge prenatal alcohol exposure and child neuropsychological outcomes: a meta-analysis. Alcohol Clin Exp Res 38:214–226. 10.1111/acer.12214 23905882

[B29] Fletcher PJ, Tampakeras M, Sinyard J, Higgins GA (2007) Opposing effects of 5-HT(2A) and 5-HT(2C) receptor antagonists in the rat and mouse on premature responding in the five-choice serial reaction time test. Psychopharmacology 195:223–234. 10.1007/s00213-007-0891-z 17673981

[B30] Fryer SL, McGee CL, Matt GE, Riley EP, Mattson SN (2007) Evaluation of psychopathological conditions in children with heavy prenatal alcohol exposure. Pediatrics 119:e733–7e741. 10.1542/peds.2006-1606 17332190

[B31] Gabbott PL, Warner TA, Jays PR, Salway P, Busby SJ (2005) Prefrontal cortex in the rat: projections to subcortical autonomic, motor, and limbic centers. J Comp Neur 492:145–177. 10.1002/cne.20738 16196030

[B32] Guillem K, Bloem B, Poorthuis RB, Loos M, Smit AB, Maskos U, Spijker S, Mansvelder HD (2011) Nicotinic acetylcholine receptor beta2 subunits in the medial prefrontal cortex control attention. Science (NY) 333:888–891. 10.1126/science.1207079 21836018

[B33] Hahn B, Sharples CG, Wonnacott S, Shoaib M, Stolerman IP (2003) Attentional effects of nicotinic agonists in rats. Neuropharmacology 44:1054–1067. 12763099 10.1016/s0028-3908(03)00099-6

[B34] Hahn B, Shoaib M, Stolerman IP (2002) Nicotine-induced enhancement of attention in the five-choice serial reaction time task: the influence of task demands. Psychopharmacology 162:129–137. 10.1007/s00213-002-1005-6 12110990

[B35] Henderson BJ, Lester HA (2015) Inside-out neuropharmacology of nicotinic drugs. Neuropharmacology 96:178–193. 10.1016/j.neuropharm.2015.01.022 25660637 PMC4486611

[B36] Hillmer AT, Tudorascu DL, Wooten DW, Lao PJ, Barnhart TE, Ahlers EO, Resch LM, Larson JA, Converse AK, Moore CF, Schneider ML, Christian BT (2014) Changes in the alpha4beta2* nicotinic acetylcholine system during chronic controlled alcohol exposure in nonhuman primates. Drug Alcohol Depend 138:216–219. 10.1016/j.drugalcdep.2014.01.027 24602361 PMC3992705

[B37] Hoover WB, Vertes RP (2007) Anatomical analysis of afferent projections to the medial prefrontal cortex in the rat. Brain Struct Funct 212:149–179. 10.1007/s00429-007-0150-4 17717690

[B38] Howe WM, Ji J, Parikh V, Williams S, Mocaër E, Trocmé-Thibierge C, Sarter M (2010) Enhancement of attentional performance by selective stimulation of alpha4beta2(*) nAChRs: underlying cholinergic mechanisms. Neuropsychopharmacology 35:1391–1401. 10.1038/npp.2010.9 20147893 PMC2855755

[B39] Infante MA, Moore EM, Nguyen TT, Fourligas N, Mattson SN, Riley EP (2015) Objective assessment of ADHD core symptoms in children with heavy prenatal alcohol exposure. Physiol Behav 148:45–50. 10.1016/j.physbeh.2014.10.014 25447751 PMC4408220

[B40] Iqbal U, Rikhy S, Dringenberg HC, Brien JF, Reynolds JN (2006) Spatial learning deficits induced by chronic prenatal ethanol exposure can be overcome by non-spatial pre-training. Neurotoxicol Teratol 28:333–341. 10.1016/j.ntt.2006.01.011 16530381

[B41] Jiang Q, Hu Y, Wu P, Cheng X, Li M, Yu D, Deng J (2007) Prenatal alcohol exposure and the neuroapoptosis with long-term effect in visual cortex of mice. Alcohol Alcohol 42:285–290. 10.1093/alcalc/agm032 17537831

[B42] Kane CJ, Phelan KD, Han L, Smith RR, Xie J, Douglas JC, Drew PD (2011) Protection of neurons and microglia against ethanol in a mouse model of fetal alcohol spectrum disorders by peroxisome proliferator-activated receptor-gamma agonists. Brain Behav Immun 25:1:S137–145. Suppl 10.1016/j.bbi.2011.02.016 21376806 PMC3104506

[B43] Kassam SM, Herman PM, Goodfellow NM, Alves NC, Lambe EK (2008) Developmental excitation of corticothalamic neurons by nicotinic acetylcholine receptors. J Neurosci 28:8756–8764. 10.1523/JNEUROSCI.2645-08.2008 18753377 PMC2909269

[B44] Kato AS, Gill MB, Yu H, Nisenbaum ES, Bredt DS (2010) TARPs differentially decorate AMPA receptors to specify neuropharmacology. Trends Neurosci 33:241–248. 10.1016/j.tins.2010.02.004 20219255

[B45] Kingdon D, Cardoso C, McGrath JJ (2016) Executive function deficits in fetal alcohol spectrum disorders and attention-deficit/hyperactivity disorder: a meta-analysis. J Child Psychol Psychiatry 57:116–131. 10.1111/jcpp.12451 26251262 PMC5760222

[B46] Koren G (2015) Pharmacological treatment of disruptive behavior in children with fetal alcohol spectrum disorder. Paediatr Drugs 17:179–184. 10.1007/s40272-015-0118-4 25634057

[B47] Korkotian E, Bombela T, Odegova T, Zubov P, Segal M (2013) Ethanol affects network activity in cultured rat hippocampus: mediation by potassium channels. PloS One 8:e75988. 10.1371/journal.pone.0075988 24260098 PMC3829821

[B48] Lambolez B, Ropert N, Perrais D, Rossier J, Hestrin S (1996) Correlation between kinetics and RNA splicing of alpha-amino-3-hydroxy-5-methylisoxazole-4-propionic acid receptors in neocortical neurons. Proc Natl Acad Sci U S A 93:1797–1802. 8700838 10.1073/pnas.93.5.1797 PMC39861

[B49] Lee KT, Mattson SN, Riley EP (2004) Classifying children with heavy prenatal alcohol exposure using measures of attention. J Int Neuropsychol Soc 10:271–277. 10.1017/S1355617704102142 15012847

[B50] Lupton C, Burd L, Harwood R (2004) Cost of fetal alcohol spectrum disorders. Am J Med Genet C Semin Med Genet 127C:42–50. 10.1002/ajmg.c.30015 15095471

[B51] Maier SE, Chen WJ, West JR (1996) Prenatal binge-like alcohol exposure alters neurochemical profiles in fetal rat brain. Pharmacol Biochem Behav 55:521–529. 8981582 10.1016/s0091-3057(96)00282-1

[B52] Majchrzak MJ, Dilsaver SC (1992) Chronic treatment with ethanol alters the physiological action of nicotine. Prog Neuro-Psychopharmacol Biol Psychiatr 16:107–115. 1557502 10.1016/0278-5846(92)90013-5

[B53] Mar AC, Horner AE, Nilsson SR, Alsiö J, Kent BA, Kim CH, Holmes A, Saksida LM, Bussey TJ (2013) The touchscreen operant platform for assessing executive function in rats and mice. Nat Protoc 8:1985–2005. 10.1038/nprot.2013.123 24051960 PMC4131754

[B54] Marquardt K, Sigdel R, Caldwell K, Brigman JL (2014) Prenatal ethanol exposure impairs executive function in mice into adulthood. Alcohol Clin Exp Res 38:2962–2968. 10.1111/acer.12577 25581651 PMC4293100

[B55] Martin D, Savage DD, Swartzwelder HS (1992) Effects of prenatal ethanol exposure on hippocampal ionotropic-quisqualate and kainate receptors. Alcohol Clin Exp Res 16:816–821. 1382392 10.1111/j.1530-0277.1992.tb00685.x

[B56] Martin G, Puig S, Pietrzykowski A, Zadek P, Emery P, Treistman S (2004) Somatic localization of a specific large-conductance calcium-activated potassium channel subtype controls compartmentalized ethanol sensitivity in the nucleus accumbens. J Neurosci 24:6563–6572. 10.1523/JNEUROSCI.0684-04.2004 15269268 PMC6729869

[B57] Martin SA, McLanahan ED, Bushnell PJ, Hunter ES, 3rd, El-Masri H (2015) Species extrapolation of life-stage physiologically-based pharmacokinetic (PBPK) models to investigate the developmental toxicology of ethanol using in vitro to in vivo (IVIVE) methods. Toxicol Sci 143:512–535. 10.1093/toxsci/kfu246 25410581

[B58] Mattson SN, Crocker N, Nguyen TT (2011) Fetal alcohol spectrum disorders: neuropsychological and behavioral features. Neuropsychol Rev 21:81–101. 10.1007/s11065-011-9167-9 21503685 PMC3410672

[B59] May PA, Blankenship J, Marais AS, Gossage JP, Kalberg WO, Joubert B, Cloete M, Barnard R, De Vries M, Hasken J, Robinson LK, Adnams CM, Buckley D, Manning M, Parry CD, Hoyme HE, Tabachnick B, Seedat S (2013) Maternal alcohol consumption producing fetal alcohol spectrum disorders (FASD): quantity, frequency, and timing of drinking. Drug Alcohol Depend 133:502–512. 10.1016/j.drugalcdep.2013.07.013 23932841 PMC3829200

[B60] McGaughy J, Decker MW, Sarter M (1999) Enhancement of sustained attention performance by the nicotinic acetylcholine receptor agonist ABT-418 in intact but not basal forebrain-lesioned rats. Psychopharmacology 144:175–182. 10.1007/s002130050991 10394999

[B61] McTighe SM, Neal SJ, Lin Q, Hughes ZA, Smith DG (2013) The BTBR mouse model of autism spectrum disorders has learning and attentional impairments and alterations in acetylcholine and kynurenic acid in prefrontal cortex. PloS One 8:e62189 10.1371/journal.pone.0062189 23638000 PMC3634761

[B62] Muir JL, Everitt BJ, Robbins TW (1996) The cerebral cortex of the rat and visual attentional function: dissociable effects of mediofrontal, cingulate, anterior dorsolateral, and parietal cortex lesions on a five-choice serial reaction time task. Cereb Cortex 6:470–481. 8670672 10.1093/cercor/6.3.470

[B63] Murphy ER, Dalley JW, Robbins TW (2005) Local glutamate receptor antagonism in the rat prefrontal cortex disrupts response inhibition in a visuospatial attentional task. Psychopharmacology 179:99–107. 10.1007/s00213-004-2068-3 15678364

[B64] Nagahara AH, Handa RJ (1997) Fetal alcohol exposure produces delay-dependent memory deficits in juvenile and adult rats. Alcohol Clin Exp Res 21:710–715. 9194928

[B65] Nagahara AH, Handa RJ (1999) Fetal alcohol-exposed rats exhibit differential response to cholinergic drugs on a delay-dependent memory task. Neurobiol Learn Mem 72:230–243. 10.1006/nlme.1999.3909 10536100

[B66] Nilsson SR, Celada P, Fejgin K, Thelin J, Nielsen J, Santana N, Heath CJ, Larsen PH, Nielsen V, Kent BA, Saksida LM, Stensbøl TB, Robbins TW, Bastlund JF, Bussey TJ, Artigas F, Didriksen M (2016) A mouse model of the 15q13.3 microdeletion syndrome shows prefrontal neurophysiological dysfunctions and attentional impairment. Psychopharmacology 233:2151–2163. 10.1007/s00213-016-4265-2 26983414 PMC4869740

[B67] O’Malley KD, Nanson J (2002) Clinical implications of a link between fetal alcohol spectrum disorder and attention-deficit hyperactivity disorder. Can J Psychiat 47:349–354. 10.1177/070674370204700405 12025433

[B68] Oesterheld JR, Kofoed L, Tervo R, Fogas B, Wilson A, Fiechtner H (1998) Effectiveness of methylphenidate in Native American children with fetal alcohol syndrome and attention deficit/hyperactivity disorder: a controlled pilot study. J Child Adolesc Psychopharmacol 8:39–48. 10.1089/cap.1998.8.39 9639078

[B69] Olmstead MC, Martin A, Brien JF, Reynolds JN (2009) Chronic prenatal ethanol exposure increases disinhibition and perseverative responding in the adult guinea pig. Behav Pharmacol 20:554–557. 10.1097/FBP.0b013e3283305e27 19633537

[B70] Olsen SR, Bortone DS, Adesnik H, Scanziani M (2012) Gain control by layer six in cortical circuits of vision. Nature 483:47–52. 10.1038/nature10835 22367547 PMC3636977

[B71] Paley B, O’Connor MJ (2009) Intervention for individuals with fetal alcohol spectrum disorders: treatment approaches and case management. Dev Disabil Res Rev 15:258–267. 10.1002/ddrr.67 19731383

[B72] Parikh V, Ji J, Decker MW, Sarter M (2010) Prefrontal beta2 subunit-containing and alpha7 nicotinic acetylcholine receptors differentially control glutamatergic and cholinergic signaling. J Neurosci 30:3518–3530. 10.1523/JNEUROSCI.5712-09.2010 20203212 PMC2864641

[B73] Parikh V, Kozak R, Martinez V, Sarter M (2007) Prefrontal acetylcholine release controls cue detection on multiple timescales. Neuron 56:141–154. 10.1016/j.neuron.2007.08.025 17920021 PMC2084212

[B74] Parikh V, Man K, Decker MW, Sarter M (2008) Glutamatergic contributions to nicotinic acetylcholine receptor agonist-evoked cholinergic transients in the prefrontal cortex. J Neurosci 28:3769–3780. 10.1523/JNEUROSCI.5251-07.2008 18385335 PMC6671097

[B75] Passetti F, Dalley JW, O’Connell MT, Everitt BJ, Robbins TW (2000) Increased acetylcholine release in the rat medial prefrontal cortex during performance of a visual attentional task. Eur J Neurosci 12:3051–3058. 10971646 10.1046/j.1460-9568.2000.00183.x

[B76] Paxinos G, Franklin KBJ (2001) The mouse brain in stereotaxic coordinates, 2nd Edition Academic: London.

[B77] Peadon E, Elliott EJ (2010) Distinguishing between attention-deficit hyperactivity and fetal alcohol spectrum disorders in children: clinical guidelines. Neuropsych Dis Treat 6:509–515. 10.2147/ndt.s7256 PMC2938300 20856914

[B78] Pedarzani P, Stocker M (2008) Molecular and cellular basis of small–and intermediate-conductance, calcium-activated potassium channel function in the brain. Cell Mol Life Sci 65:3196–3217. 10.1007/s00018-008-8216-x 18597044 PMC2798969

[B79] Poorthuis RB, Bloem B, Schak B, Wester J, de Kock CP, Mansvelder HD (2013) Layer-specific modulation of the prefrontal cortex by nicotinic acetylcholine receptors. Cereb Cortex 23:148–161. 10.1093/cercor/bhr390 22291029 PMC3513956

[B80] Popova S, Lange S, Burd L, Rehm J (2016) The economic burden of fetal alcohol spectrum disorder in Canada in 2013. Alcohol Alcohol 51:367–375. 10.1093/alcalc/agv117 26493100

[B81] Quarta D, Naylor CG, Morris HV, Patel S, Genn RF, Stolerman IP (2007) Different effects of ionotropic and metabotropic glutamate receptor antagonists on attention and the attentional properties of nicotine. Neuropharmacology 53:421–430. 10.1016/j.neuropharm.2007.05.023 17631918

[B82] Reyes E, Wolfe J, Savage DD (1989) The effects of prenatal alcohol exposure on radial arm maze performance in adult rats. Physiol Behav 46:45–48. 2813555 10.1016/0031-9384(89)90319-3

[B83] Riley EP, Infante MA, Warren KR (2011) Fetal alcohol spectrum disorders: an overview. Neuropsychol Rev 21:73–80. 10.1007/s11065-011-9166-x 21499711 PMC3779274

[B84] Robbins TW (2002) The 5-choice serial reaction time task: behavioural pharmacology and functional neurochemistry. Psychopharmacology 163:362–380. 10.1007/s00213-002-1154-7 12373437

[B85] Robles N, Sabriá J (2008) Effects of moderate chronic ethanol consumption on hippocampal nicotinic receptors and associative learning. Neurobiol Learn Mem 89:497–503. 10.1016/j.nlm.2008.01.006 18331803

[B86] Roozen S, Peters GJ, Kok G, Townend D, Nijhuis J, Curfs L (2016) Worldwide prevalence of fetal alcohol spectrum disorders: a systematic literature review including meta-analysis. Alcohol Clin Exp Res 40:18–32. 10.1111/acer.12939 26727519

[B87] Ryan SH, Williams JK, Thomas JD (2008) Choline supplementation attenuates learning deficits associated with neonatal alcohol exposure in the rat: effects of varying the timing of choline administration. Brain Res 1237:91–100. 10.1016/j.brainres.2008.08.048 18786517 PMC2646103

[B88] Sherman SM (2016) Thalamus plays a central role in ongoing cortical functioning. Nat Neurosci 16:533–541. 10.1038/nn.4269 27021938

[B89] Smiley JF, Saito M, Bleiwas C, Masiello K, Ardekani B, Guilfoyle DN, Gerum S, Wilson DA, Vadasz C (2015) Selective reduction of cerebral cortex GABA neurons in a late gestation model of fetal alcohol spectrum disorder. Alcohol (Fayetteville) 49:571–580. 10.1016/j.alcohol.2015.04.008 PMC4554880 26252988

[B90] Snyder J, Nanson J, Snyder R, Block G (1997) A study of stimulant medication in children with FAS. In: Challenge of Fetal Alcohol Syndrome, University of Washington Press: Seattle, 64–77.

[B97] Tian MK, Bailey CD, Lambe EK (2014) Cholinergic excitation in mouse primary vs. associative cortex: region-specific magnitude and receptor balance. Eur J Neurosci 40:2608–2618. 24827827 10.1111/ejn.12622 PMC4640901

[B91] Sokol RJ, Delaney-Black V, Nordstrom B (2003) Fetal alcohol spectrum disorder. JAMA 290:2996–2999. 10.1001/jama.290.22.2996 14665662

[B92] Wada E, McKinnon D, Heinemann S, Patrick J, Swanson LW (1990) The distribution of mRNA encoded by a new member of the neuronal nicotinic acetylcholine receptor gene family (alpha 5) in the rat central nervous system. Brain Res 526:45–53. 2078817 10.1016/0006-8993(90)90248-a

[B93] West JR, Goodlett CR, Bonthius DJ, Pierce DR (1989) Manipulating peak blood alcohol concentrations in neonatal rats: review of an animal model for alcohol-related developmental effects. Neurotoxicology 10:347–365. 2696896

[B94] Woolfrey KM, Hunt PS, Burk JA (2005) Postnatal ethanol exposure disrupts signal detection in adult rats. Neurotoxicol Teratol 27:815–823. 10.1016/j.ntt.2005.07.002 16115748

[B95] Zikopoulos B, Barbas H (2006) Prefrontal projections to the thalamic reticular nucleus form a unique circuit for attentional mechanisms. J Neurosci 26:7348–7361. 10.1523/JNEUROSCI.5511-05.2006 16837581 PMC6674204

[B96] Zuo Y, Nagata K, Yeh JZ, Narahashi T (2004) Single-channel analyses of ethanol modulation of neuronal nicotinic acetylcholine receptors. Alcohol Clin Exp Res 28:688–696. 15166642 10.1097/01.alc.0000125349.99823.8a

